# Evaluation of electrostatic sprayers and foggers for the application of disinfectants in the era of SARS-CoV-2

**DOI:** 10.1371/journal.pone.0257434

**Published:** 2021-09-30

**Authors:** Joseph P. Wood, Matthew Magnuson, Abderrahmane Touati, Jerome Gilberry, Jonathan Sawyer, Timothy Chamberlain, Stella McDonald, David Hook

**Affiliations:** 1 Office of Research and Development, United States Environmental Protection Agency, Research Triangle Park, North Carolina, United States of America; 2 Office of Research and Development, United States Environmental Protection Agency, Cincinnati, Ohio, United States of America; 3 Jacobs Technology, Inc., Research Triangle Park, North Carolina, United States of America; University of Manitoba, CANADA

## Abstract

Although research has shown that the COVID-19 disease is most likely caused by airborne transmission of the SARS-CoV-2 virus, disinfection of potentially contaminated surfaces is also recommended to limit the spread of the disease. Use of electrostatic sprayers (ESS) and foggers to rapidly apply disinfectants over large areas or to complex surfaces has emerged with the COVID-19 pandemic. ESSs are designed to impart an electrostatic charge to the spray droplets with the goal of increasing deposition of the droplets onto surfaces, thereby promoting more efficient use of the disinfectant. The purpose of this research was to evaluate several spray parameters for different types of sprayers and foggers, as they relate to the application of disinfectants. Some of the parameters evaluated included the spray droplet size distribution, the electrostatic charge, the ability of the spray to wrap around objects, and the loss of disinfectant chemical active ingredient due to the spray process. The results show that most of the devices evaluated for droplet size distribution had an average volume median diameter ≥ 40 microns, and that four out of the six ESS tested for charge/mass produced sprays of at least 0.1 mC/kg. A minimal wrap-around effect of the spray deposition onto a cylindrical object was observed. The loss of disinfectant active ingredient to the air due to spraying was minimal for the two disinfectants tested, and concurrently, the active ingredient concentrations of the liquid disinfectants sprayed and collected 3 feet (1 meter) away from the spray nozzle do not decrease.

## Introduction

Recent research has shown that the Coronavirus Disease 2019 (COVID-19) is most likely caused by airborne transmission of the Severe Acute Respiratory Syndrome Coronavirus-2 (SARS-CoV-2), but it is believed that the virus may also be transmitted via the contact of contaminated surfaces [1]. Thus, disinfection of potentially contaminated surfaces is recommended, among other activities, to limit the spread of the disease [2]. Use of electrostatic sprayers (ESS) and foggers to rapidly apply disinfectants over large areas or to complex, intricate surfaces has increased substantially with the COVID-19 pandemic.

ESS have been used for many years in several other industries, e.g., for the efficient application of pesticides to crops (to reduce spray drift) [3–5] and vegetation [6]. Recently ESS have emerged as an application technique for disinfectants for surfaces potentially contaminated with the SARS-CoV-2 virus [7].

ESSs are designed to impart an electrostatic charge to the spray droplets (most do so as the droplets exit the nozzle of the sprayer), with the goal of increasing deposition of the droplets onto surfaces, thereby promoting more efficient use of the disinfectant with less waste. Further, electrostatics may enhance the uniformity of the spray, and increase bio-efficacy and droplet adhesion [8]. The use of an ESS may allow less disinfectant to be used to cover a surface area, but with less disinfectant applied, disinfection efficacy may diminish if the surface does not remain wet for the required contact time.

The electrostatic spray process is complicated and involves multiple physical phenomena, including aerosol droplet generation, fluid dynamics, and electrostatics [8]. Hence there are several spray parameters that may impact a disinfectant’s ability to inactivate the virus on surfaces, notwithstanding that an ESS is only as effective as the disinfectant chemical being sprayed (in the United States, only Environmental Protection Agency-approved disinfectants should be used for the SARS-CoV-2 virus and done so in accordance with their label) [9].

The following parameters were evaluated in this study; note that some of these are relevant to any type of powered-sprayer or fogger in which large areas are being disinfected and include the following:

- The amount of disinfectant to apply to a surface, or in other words, the surface coverage or deposition (in terms of fluid volume of disinfectant per unit area), so that the surface remains wet for the required contact time to ensure inactivation of the virus.
- The electrostatic charge imparted to the spray, potentially enhancing its ability to deposit onto and adhere to surfaces, including surfaces not in the direct path of the spray, e.g., the ability to wrap around and adhere to complex surfaces. Many manufacturers and proponents of ESS tout this ability.
- The amount of the disinfectant’s active ingredient (AI) lost to the air before reaching the surface. Loss of AI to the air may diminish its concentration on the surface, thus potentially reducing disinfection efficacy.
- The droplet size distribution of the spray. Smaller droplets (e.g., < 100 microns) may tend to drift off-target more but may be more effective per unit mass as pesticides or fungicides; electrostatics can be used to control droplet trajectories [10].

Two of the above parameters are also related to exposure concerns by creating inhalation hazards to the operator of the ESS or those occupying the space following disinfection. The droplet size distribution of the spray and chemical composition of the droplets are of concern, since smaller droplets are more readily inhaled and deposit deeper in the respiratory tract. The loss of AI of the disinfectant to the vapor phase during the spray process is also a concern. Some disinfectant AI chemicals, such as ethanol, chlorine, and hydrogen peroxide, may volatilize and become hazardous at sufficiently high vapor concentrations in enclosed spaces or localized area around the ESS operation.

The purpose of this research was to evaluate the aforementioned spray parameters for different types of sprayers and foggers, as they relate to the application of disinfectants. Several studies have confirmed that an ESS is a suitable technique for the application of disinfectants to effectively inactivate bacteria and viruses [11–14], but without investigating underlying ESS parameters. Other studies have documented their use for effective application of a decontaminant against a chemical agent [15] and a *Bacillus anthracis* (causative agent for anthrax disease) spore-forming surrogate [16], while dispensing less liquid. Several studies have investigated their use in agricultural pesticidal applications, for example, Sasaki et al. [17], have investigated spray deposition as a function of electrostatic charge. To the best of our knowledge, this is the first study to examine several physical parameters for several ESS and other devices as they relate to application of disinfectants in an indoor environment.

## Materials and methods

Six ESS, two foggers, and one hand-pumped garden sprayer were evaluated in the study (Table 1). The devices were selected for our study based upon an initial assessment of commercial availability.

**Table 1:** Summary of spray and fog devices evaluated in the study.

	Manufacturer or distributor	Type of device	Source of electrical power	Notes
**PX200ES handheld (HH)**	Earthsafe Chemical Alternatives, Braintree, MA	ESS	Battery	This model has the ability to turn on and off the electrostatics. The Li ion battery for this device was later recalled.
**PX300ES backpack**	Earthsafe Chemical Alternatives, Braintree, MA	ESS	Battery	This sprayer came with a 40-micron (red) and 80-micron (green) nozzle. The Li ion battery for this device was later recalled.
**SC-ET**	Electrostatic Spraying Systems, Watkinsville, GA	ESS	Alternating current (cord plug-in)	Purchased in ~ 2015 and used in several US EPA studies over the years, prior to this study. All the other devices evaluated were newly purchased for this study.
**EM360 HH**	Emist, Fort Worth, TX	ESS	Battery	
**R40**	360 Sterile, Burnaby, BC, CA	ESS	Battery	Lithium ion battery failed and was later replaced
**Total 360**	ByoPlanet, for Clorox, Oakland, CA	ESS	Alternating current (cord plug-in)	
**Professional Sprayer 2-gallon R20S16**	Husqvarna, Charlotte, NC	garden sprayer	No electrical; hand pumped	
**Airofog Flex ULV cold fogger U120**	Airofog USA, Brooksville, FL	fogger	Alternating current (cord plug-in)	
**Mist Duster KB-15002E 12L**	Ipihsius via Amazon.com	fogger	Alternating current (cord plug-in)	This device was not tested for spray charge due to it becoming non-functioning during the droplet size distribution tests.

Sprayers are used to apply disinfectant directly to a surface (recommended spray distances vary from about 2 feet to 10 feet [0.6–3.0 m]), whereas foggers may be used for disinfection or decontamination of both surfaces and volumes (i.e., disinfection of air, inactivation of aerosolized viral particles) [18]. Because the disinfectant chemical fog can fill a room, they are usually operated automatically with no operator present [18]. The two foggers we evaluated in this study do not use electrostatic charging of their droplets.

One of the ESS in our study came with two different nozzles, stated to produce different size droplets (a 40 micron and 80-micron volumetric median diameter, or VMD), and thus both nozzles were evaluated. With another ESS, the operator can switch the electrostatic charge on and off, and so both settings were evaluated for some of the parameters.

Both water and disinfectants were tested in the sprayers, albeit the latter to a lesser extent. Finally, we note that some of the sprayers were malfunctioning at the time certain parameters were being evaluated, and so not all were tested for every parameter; the lithium ion batteries were an issue for two of the ESS.

The primary spray or fog device parameters evaluated in the study included the droplet size distribution, the electrostatic charge, and the spray deposition (wrap-around effect). We also evaluated the fate of the active ingredient for two disinfectants during the spray process. Lastly, we reviewed the manufacturer supplied literature for their recommended disinfectant surface coverage or deposition amount.

### Sprayer manufacturer-recommended surface coverage or deposition

This parameter is critical to ensure that sufficient disinfectant volume is applied to the surface such that it remains wet for the required contact time of the disinfectant, and thus ensures effective inactivation of the virus (and in compliance with the disinfectant label [19]). This parameter was compiled from the sprayer user manuals, brochures, and distributor/manufacturer websites. Since ESSs are typically used for disinfection of large surface areas, the suggested deposition amount is usually presented in units of fluid ounces of disinfectant per 1000 ft^2^ (1 oz/1000 ft^2^ = 0.3 ml/m^2^). As previously discussed, foggers are primarily used for volumetric decontamination, and so no suggested surface coverage rate was provided for the two foggers we evaluated. (For the foggers, no vendor-recommended amount of disinfectant per unit volume of space to be disinfected was provided either, since this would be dependent on the target microorganism and the disinfectant being fogged, among other parameters.) This parameter is presented here to provide the reader with an indication of the range in values as suggested by the manufacturers.

### Sprayer flow rate

Each sprayer’s flow rate was evaluated for 30 seconds by spraying into a large container and then transferring the liquid to a graduated cylinder for measurement. Sprayer flow rates were measured in conjunction with other tests (e.g., measuring the spray droplet size distribution), and these are reported here. The sprayer was running for a few seconds before placing the nozzle in the container and starting the timer. With the flow rate known, the time required to dispense the disinfectant onto a surface can be calculated as follows:

$$T\;=\;\left(A\times D\right)÷Q$$

Where T = time

A = total surface area to be disinfected (m^2^)

D = deposition (mL/m^2^)

Q = flow rate (mL/min)

### Droplet size distribution of the spray

The droplet size distribution of a spray is typically characterized in terms of the VMD, which refers to the droplet size in which half the volume of the spray is in droplets less than, and half of the volume of the spray is in droplets greater than, the VMD. It is typically reported in units of microns. Results for the droplet size distribution may also be reported in terms of other percentages for which the volume of the spray is less than the specified diameter. Examples include the Dv10 or Dv90, which is the droplet diameter in which 10% or 90% of the volume of the spray is less than that droplet size, respectively. These measurements provide additional characterization of the size range of the spray droplets than the VMD alone.

**Fig 1:**
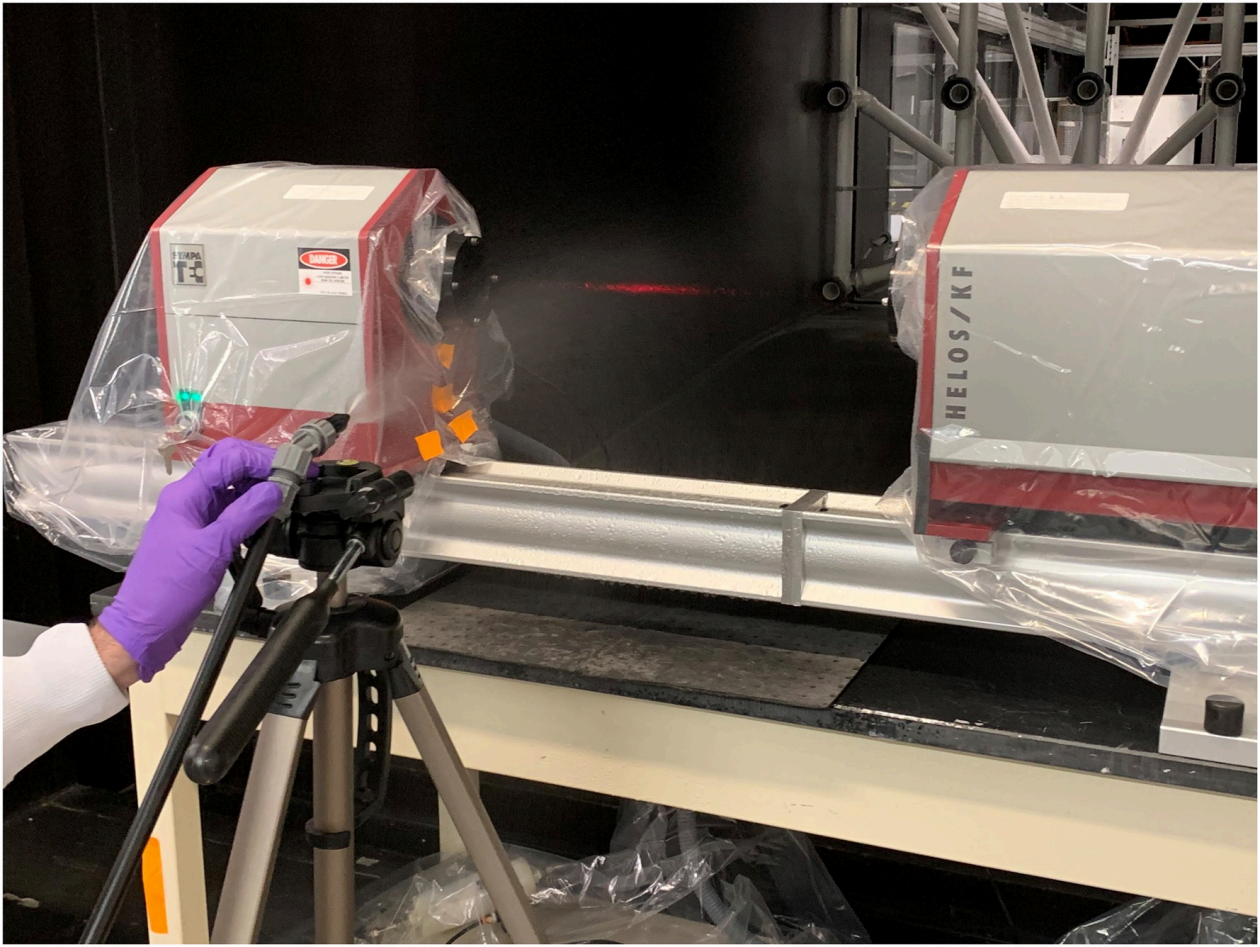
Photograph of a spray cone being directed into the optical path of the laser diffraction instrument. Note resulting illumination of the red laser beam.

Data were collected for analysis of the volume-based droplet size distributions using a forward scattering laser diffraction instrument (HELOS/KR-Vario; Sympatec GmbH, Clausthal-Zellerfeld, Germany) using methods similar to a previous study [20]; refer to Fig 1 for a photograph of the instrument in operation with an ESS. The instrument was placed in a recirculating wind tunnel and the tunnel was set to 20°C (±1°C) and 50% (±5%) relative humidity (RH). A description of the EPA’s aerosol wind tunnel, its control system and instrumentation, are described here [21]. Each of the sprayers’ droplet size distributions were measured with at least one distance within the range recommended by the manufacturer (if provided). Other distances were selected for comparative analysis or were beyond the recommended range to determine impact. For some sprayers/distances, the spray density was too great and produced multiple scattering in the instrument, and so the sprayer had to be moved back. Prior to each measurement, the wind tunnel fan was turned off so that there was no airflow. At each spray distance, the droplet size distribution was analyzed five times with the spray perpendicular to the laser. The droplet size distribution of the sprayers was measured using deionized water as well as laboratory-acquired tap water.

Following the tests with water, the droplet size distribution for one ESS was evaluated for three different water-based disinfectants to assess the impact the presence of the disinfectant solution might have on the droplet size distribution. The three disinfectants evaluated utilized an AI of either chlorine (specifically, sodium dichloro-s-triazinetrione, or “dichlor”), hydrogen peroxide, or quaternary ammonium. However, when testing the quat-based disinfectant, the spray penetrated the sheath air protecting the optics of the instrument and thus coated the lenses, rendering the data for this disinfectant unusable. The disinfectants were prepared according to the label directions.

Prior to the above tests, the HELOS calibration was verified to determine if there were any issues with the optics not being installed correctly or the lenses being dirty. Test particles of glass beads (#18901–100, Polysciences, Inc., Warrington, PA) nominal 30–50-micron diameter were sifted through a 60-micron sieve and fell under gravity through the HELOS laser optical path 5 times. The D50 and VMD were calculated and averaged to be both 41 microns. Through these checks it was verified that the particle size distribution was relatively gaussian (D50 and VMD having similar values) and within 10% of the nominal value of the glass beads (40 microns).

### Electrostatic charge of the spray

The test apparatus used to measure electrostatic charge of the sprays was custom-built and based in part on a previous study [22]. The measurement device consisted of an aluminum plate with dimensions of 51 cm by 29 cm, mounted to plywood of similar dimensions using zinc screws at each corner. Two holes were bore at the top of the plate and rubber screen spline was used to suspend the plate at a height of 132 cm (to center point) from the floor in the center of the wind tunnel. The average wind tunnel temperature and RH measured approximately 23°C and 47.0%, respectively. A picoammeter (Keithley model 4145; Tektronix, Inc., Beaverton, OR) was used to directly measure the current generated from spraying the plate with electrostatically charged droplets and was connected to the top corner of the plate via positive lead with an alligator clip. The other lead was sent to ground via a ground plug to a wall receptacle.

Each sprayer was sprayed directly at the plate from a one-foot distance, using a sweeping motion to fully wet the plate over the course of 30 seconds, while recording the electrical current. This process was repeated three times with the plate being wiped dry between each test. After three measurements were collected, each sprayer was evaluated for flow rate. The charge/mass ratio was determined by calculating the average current measured from the three tests divided by the mass flow rate. The liquid flow rate was converted to mass flow rate by multiplying by the density of water (1 g/mL). The charge/mass ratio results are reported in units of millicoulomb/kg. All tests were conducted with the ESS operator wearing an insulator mitt, and all ESS were operated according to the manufacturers’ instructions provided, related to the use of any grounding requirements.

The electrostatic charge imparted to the droplets from the sprayers was measured for both tap water and deionized water, to determine if the presence of ions (which may alter the conductivity) had any effect on the spray charge. Charge measurements were conducted for all the devices, except for one of the foggers, which was not functioning (and could not be fixed) during the time the tests took place. As with the droplet size distribution measurements, the spray charge was measured for both the “on” and “off” positions for one of the ESS, as well as for both nozzle tips for another ESS.

Following the tests using water, the spray charge for one of the ESS was evaluated for the same three disinfectants used in the droplet size distribution tests to assess the impact the presence of the disinfectant might have on the charge.

### Spray deposition (wrap-around effect)

This series of tests was conducted to qualitatively assess, and document with photographs, the ability of the spray (electrostatic or not) or fog to wrap around and deposit on a cylindrical object. The methods described here are based in part on discussions and information provided by ByoPlanet International (Athens, GA), an ESS manufacturer.

A metal trash can (11 inch height, 8 inch diameter [30 cm by 3.1 cm] with a black matte finish was initially used in the study, and then followed with a few additional tests to examine the spray deposition on objects with more complicated surfaces, such as a step ladder, a clip-on lamp, and fold-out chair. These tests were conducted in EPA’s COMMANDER test chamber, described elsewhere [23].

The devices were filled with an aqueous solution of fluorescent dye (Blue aqueous tracer, T-900, Black Light World), at a dilution of 1:25 in tap water. (For the Total 360 sprayer, we used both tap and DI water as the diluent in these tests, to evaluate whether the lack of ions in the water affected deposition.) In each test, the spray nozzle was placed at the same height as the center of the can and was placed at a distance from the can based on the owner’s manual for the sprayer. Each sprayer was evaluated using three replicate trash cans (i.e., each can was sprayed separately). A 3-inch by 3-inch square was marked on each can at 90-degree intervals using a UV-A fluorescent pen, and labeled as front, back, left and right. We targeted the spray time so that approximately 8 mL ± 1 mL were dispensed in each spray test. During each spray, the sprayer was moved back and forth, so that the spray cone fully enveloped the trash can.

Following each spray, the overhead lights in the chamber were turned off, and two, 24-inch long black lights (ADJ Products; 24BLB) were placed in front of the can to observe the deposition of the fluorescent aqueous mixture. Digital photographs (Canon Powershot SX710HS; flash off) were then taken of the front quadrant of the can, and then the can was rotated in 90-degree increments and photographs were taken for each quadrant. At each quadrant, the camera was placed directly in front of the can at two different distances, so that photos were taken of the whole can, and then up close for each square.

Before spraying the trash cans (and the other objects), photographs were taken of each can as described above, to serve as controls. Following each sprayer evaluation, the three trash cans were washed with a laboratory detergent (Alconox), and then further cleaned using a mixture of isopropyl alcohol and acetic acid.

### Fate of disinfectant AI from spraying

Measurements of the disinfectant AI for both the vapor and liquid phase were conducted to assess the fate of the AI during the spray process. In this limited set of tests, one ESS (PX200ES) was used to spray two different disinfectants in the test chamber (the air flow in the chamber shut off). We measured chlorine gas when spraying a dichlor-based disinfectant and hydrogen peroxide vapor when spraying a hydrogen peroxide-based disinfectant. We also measured the AI concentrations in the liquid phase for the disinfectants, at four stages of the spray process: the disinfectant as prepared, after filling the sprayer reservoir, when collected directly from the spray nozzle, and when collected 3 feet (1 m) away from the spray nozzle in 1-liter glass beakers. When collecting the disinfectant droplets in the glass beakers, the spray time was typically 1.5 minutes. Three replicate spray tests were conducted for each disinfectant. In all tests the disinfectants were prepared with tap water and as directed on the label.

Measurement of chlorine gas and hydrogen peroxide vapor were conducted in real-time using electrochemical sensors (Analytical Technologies, Inc., Collegeville, PA; model B12-34-1-0100-1 for hydrogen peroxide vapor and model B12-11-6-0200-1 for chlorine gas). The gas sensor was suspended from the ceiling in the center of the chamber, approximately 3 feet from the sprayer nozzle.

The AI concentration of the disinfectants in the liquid phase was measured using wet chemistry titration techniques. Free available chlorine (FAC; as hypochlorite/hypochlorous acid and combined from dichlor) was measured when using the dichlor-based disinfectant (Hach Method 10100, model CN-HRDT, Hach, Loveland, CO) which was adapted from ASTM Method D2022-89. For measuring the hydrogen peroxide concentration in the disinfectant solutions, 5 g of sample was transferred to a 250-mL flask, then 40 mL of sulfuric acid and 150 mL of deionized (DI) water were added. The sample was then titrated to a permanent pink with 1N KMnO_4_. The volume of KMnO_4_ to reach the endpoint was used to calculate the percent by weight of hydrogen peroxide.

### Statistical analysis

Differences in average results for certain parameters were reported to be significant based on t-tests calculating p-values ≤ 0.05. The p-values were calculated using MS Excel for certain results from the droplet size distribution (VMD) and electrostatic charge tests (charge/mass). Sample size was 5 for each device/distance/fluid in the droplet size tests and the sample size was 3 for the charge/mass measurements for each device/fluid combination.

## Results and discussion

### Recommended surface coverage

The manufacturer-recommended disinfectant surface coverage ranged from approximately 1–13 ml/m^2^ (2–53 ounces of disinfectant per 1000 ft^2^) for the ESS for which information was available (Table 2). No surface coverage quantities were recommended for the two foggers, consistent with the approach that they are typically intended to be used as a volumetric decontamination device rather than strictly for application of the disinfectant to surfaces. The manufacturer for the SC-ET model did not provide a surface coverage but rather recommended that a wetness test be conducted to determine the proper coverage amount such that the surface remains wet with the disinfectant for the required contact time. This approach was utilized since it is imperative that the surface remains wet for the required contact time [24] as per the disinfectant label requirements, to ensure the virus is effectively inactivated.

**Table 2:** Summary of device flow rates and recommended surface coverage.

	Average flow rate ± SD mL/min	Manufacturer-recommended surface coverage (mL/m^2^)
**PX200 ES on** ^1^	113 ± 6	12.7
**PX200 ES off** ^1^	113 ± 4	12.7
**PX300 (red nozzle)** ^2^	113 ± 2	8.9
**PX300 (green nozzle)** ^2^	140 ± 4	8.9
**SC-ET** ^3^	104 ± 5	NA^4^
**EM360**	60 ± 2	0.6
**R40**	180 ± 3	16.9
**Total 360**	122 ± 5	4.5
**Garden sprayer**	509 ± 22	NA
**Airofog**	133 ± 5	NA
**KB-15002E**	333 ± 6	NA

Notes:

NA = not available or applicable; SD = standard deviation.

^1^.This model has the ability to turn on and off the electrostatics.

^2^.This device had different nozzles to adjust droplet size.

^3^.This device was purchased in ~ 2015 and used in several previous studies, prior to this study. All the other devices evaluated were newly purchased for this study.

^4^. Manufacturer recommends a wetness test to determine coverage.

### Flow rate

Most of the sprayers and foggers evaluated had flow rates in the range of approximately 100–200 mL/min (1.6–3.2 gallon/hr; Table 2). The hand pumped garden sprayer and one of the foggers had the highest average flow rates, at 509 and 333 mL/min, respectively. The lowest flow rate observed was for the EM360 model, at 60 mL/min; refer to Table 2. Using this flow rate as an example, a surface area of 1000 m^2^ to be disinfected with a coverage of 9 mL/m^2^, would require 2.5 hr of spray time.

### Droplet size distribution

Table 3 provides a summary of the droplet size distribution measurement results, in terms of the average VMD values obtained for all sprayer configurations, spray distances, and sources of water tested. The results shown are the average VMD values for the five measurements taken at each distance. Graphs of the cumulative size distributions of the sprays for each device and source of water provide further visual detail of their droplet size distributions and are found in S1 File.

**Table 3:** Average volume median diameter for spray devices tested with deionized and tap water.

Sprayer	Spray distances (ft) evaluated	Vendor- recommended spray distance (ft)	VMD (microns) DI water	VMD (microns) Tap water
PX200 ES on	2	3–6	72 ± 1.6	78 ± 8.3
	3		69 ± 4.6	62 ± 3.1
	6		36 ± 3.9	37 ± 1.5
PX200 ES off	2		86 ± 0.9	72 ± 3.1
	3		77 ± 1.5	61 ± 4.1
	6		38 ± 2.1	38 ± 2.6
PX300 red nozzle	3	2–3	54 ± 3.8	61 ± 3.1
	5		45 ± 3.1	46 ± 1.1
	6		38 ± 1.4	41 ± 2.4
PX300 green nozzle	3		53± 2.3	51 ± 1.0
	5		44 ± 1.3	35 ± 1.6
	6		39 ± 2.1	32 ± 0.9
SC-ET	6	Up to 8 feet	25 ± 0.5	26 ± 0.4
	8		27 ± 0.4	26 ± 0.5
	10		27 ± 0.5	28 ± 0.5
EM360	3	2–3	101 ± 2.7	92 ± 1.7
	5		89 ± 2.9	91 ± 3.5
	6		80 ± 1.9	83 ± 3.3
R40	3	Not provided	68 ± 3.2	71 ± 0.9
	4		NA	55 ± 1.9
	5		50 ± 5.5	42 ± 2.6
	6		42 ± 2.2	NA
Total 360	4	2–4	45 ± 0.6	33 ± 0.5
	6		35 ± 1.2	42 ± 1.4
	8		41 ± 1.5	40 ± 0.6
Garden sprayer	3	Not provided	207 ± 3.5	174 ± 14
	5		NA	180 ± 252
	6		159 ± 37	49 ± 2.7
	10		59 ± 2.0	NA
Airofog	3	Minimum 3 feet	39 ± 0.4	40 ± 0.6
	6		42 ± 0.6	43 ± 0.6
	10		44 ± 1.2	43 ± 0.5
KB-15002E	10	15–20	40 ± 0.4	NA
	15		41 ± 0.3	NA

VMD = volume median diameter; NA = Not available (device became non-functioning after tests with DI water); NT = not tested at that spray distance.

The majority of the devices’ total spray volumes ranged with droplet sizes between 10–100 microns, and with an average VMD ≥ 40 microns. (Refer to the cumulative size distributions in S1 File and Table 3.) The SC-ET had average VMDs ranging from 25–27 microns, was purchased in 2015, and has been used in several past and current US EPA studies. These VMD results are consistent with current regulatory guidance on the use of ESS for the application of disinfectants, which limit the VMD to be ≥ 40 microns [19]. Droplet size distributions for the two foggers were in the range of the six ESS. Not surprisingly, the garden sprayer generally had larger droplets, with its maximum average VMD at 207 microns. Otherwise, the average VMDs of the devices were all ≤ 101 microns. Excluding the garden sprayer, the average DV10 values for the devices ranged from approximately 10–50 microns, and the average DV90 values ranged from approximately 50–165 microns; refer to Supporting Information. The average VMD for the ESS generally decreased with spray distance, presumably due to the larger droplets falling out before reaching the optical path of the droplet size measurement instrument. In contrast, the average VMD of the two foggers increased with spray distance, although the increase was only a few microns.

For the PX200 device in which the electrostatic charge could be turned on and off, there was no significant difference in the VMD for these two modes of operation. Unexpectedly, there was no significant difference in the VMD for the PX300 device with the two different nozzle sizes, when spraying DI water. And for this same ESS when spraying tap water, its red nozzle (manufacturer-provided information indicated droplet size would be 40 micron) produced a significantly larger (> 9–10 microns) VMD compared to the green nozzle (80 micron). The p-values were ≤ 0.005 for the three comparisons (three-, five-, and six-foot spray distances) made between the two nozzles. We are uncertain how to explain this, other than it is unclear how ESS manufacturers measure the droplet size distributions of their sprayers.

The test results also showed that the lack of ions in the water had no significant effect on the droplet size distribution (i.e., comparing deionized with tap water), as expected. Out of 28 available comparisons that could be made for the VMDs when using DI vs. tap water, the VMD was higher for the tap water in 13 of those cases; the VMDs were equivalent in one case; and the VMD was higher for DI water in 14 of the cases.

**Fig 2:**
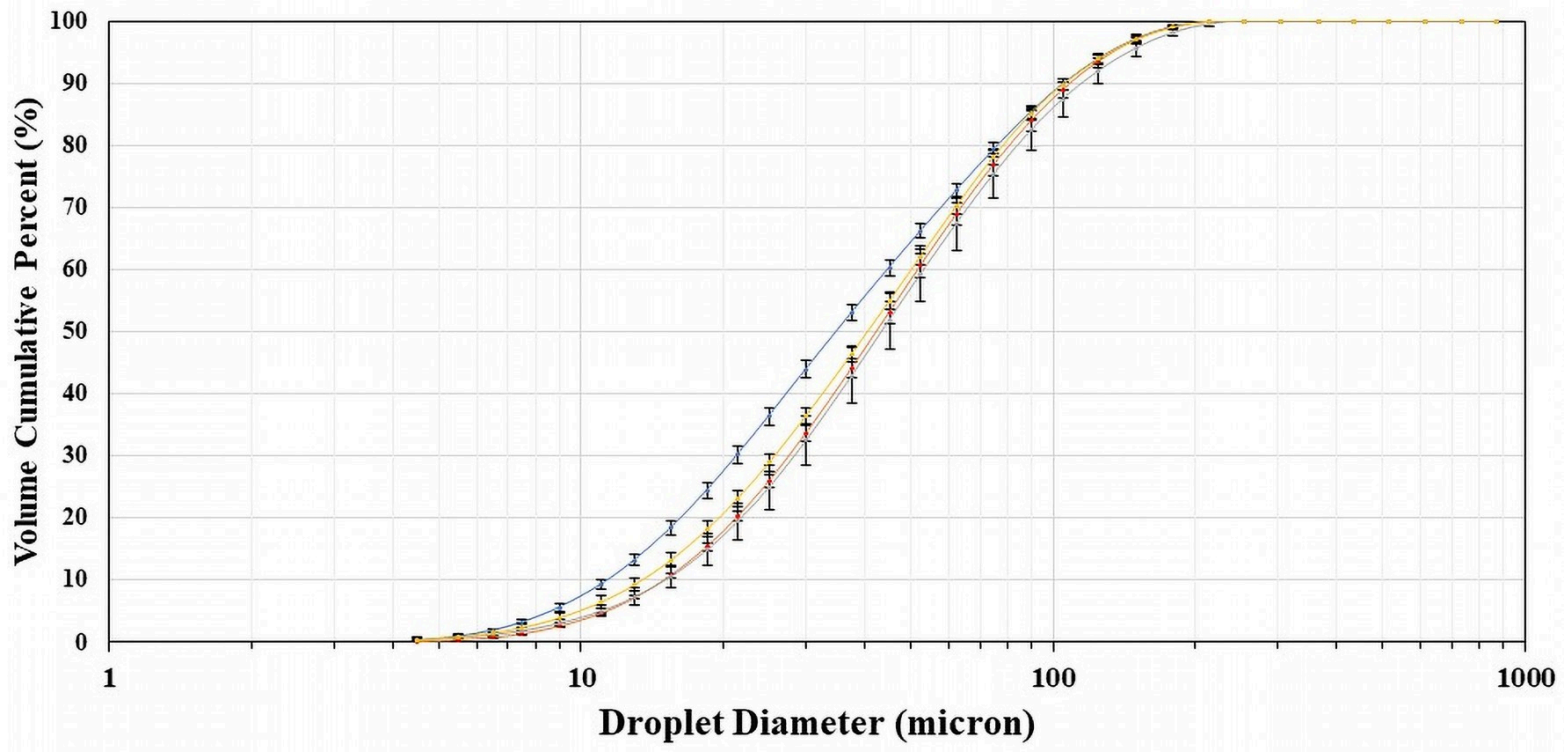
Cumulative size distributions by volume for the total 360 sprayer, at 6-foot distance, when spraying different liquids. Blue line = deionized water; red line = tap water; Grey line = disinfectant with dichlor as the active ingredient; Yellow line = disinfectant with hydrogen peroxide as its active ingredient.

The use of the two disinfectants also did not affect the droplet size distribution. Refer to Fig 2 which shows the cumulative size distribution data taken at a spray distance of 6 feet, for the two waters and two disinfectants. (Refer to the S1 File for the cumulative size distributions for all spray distances evaluated for the disinfectants.) We acknowledge that our testing only included one ESS (the Total 360) to assess the effect of disinfectant chemistry on droplet size distribution, and that other sprayers’ droplet size distributions may be differently impacted by these same disinfectants. However, this is unlikely since the disinfectants that we used in these tests were more than 99.5% water. Nevertheless, ESSs should be evaluated in conjunction with the specific disinfectant being applied, i.e., spray parameters should be evaluated for a specific ESS/disinfectant/environmental system.

### Electrostatic charge

A summary of the electrostatic charge results, when spraying either deionized or tap water, is shown in Table 4. As expected, the fogger and hand-pumped garden sprayer showed no measurable electrostatic charge. Unexpectedly, one of the ESS also showed no measured charge (R40). The two ESS that utilize alternating current (the SC-ET and the Total 360) demonstrated the highest average charge/mass (approximately -3.6 to -6.0 mC/kg), as well as their sprays having a negative charge. The sprays from the battery-powered ESSs all carried a positive electrostatic charge and were about an order of magnitude lower in charge/mass compared to the SC-ET sprayer. All of the charge/mass data included in the literature that were reviewed for this study (all were for ESS used for agricultural pesticide applications) were of positive polarity, most likely to take advantage of the net negative charge on the Earth’s surface [25].

**Table 4:** Charge/mass ratio for sprayers.

Sprayer	Average Charge to Mass Ratio, mC/kg DI water	Average Charge to Mass Ratio, mC/kg Tap water
PX200 ES on	0.109 ± 0.00	0.134 ± 0.03
PX200 ES off	0.005 ± 0.00	0.004 ± 0.00
PX300 red	0.049 ± 0.00	0.053 ± 0.00
PX300 green	0.045 ± 0.00	0.049 ± 0.00
Total 360*	-6.05 ± 0.09	-5.74 ± 0.20
EM360	0.28 ± 0.00	0.29 ± 0.01
SC-ET*	-3.56 ± 0.22	-3.28 ± 0.06
R40	0.00	0.00
Garden sprayer	0.00	0.00
Airofog	0.00	0.00
Total 360* HP	-1.79 ± 0.06	
Total 360* Quat	-1.08 ± 0.06	
Total 360* dichlor	-1.53 ± 0.00	

DI = deionized water; Tap = tap water; HP = hydrogen peroxide-based disinfectant; Quat = quaternary ammonium-based disinfectant; dichlor = dichlor-based disinfectant. The KB-15002E fogger was not functioning during spray charge measurements.

*These ESS utilize alternating current.

It is not known what magnitude of charge/mass is necessary to elicit benefits of electrostatic deposition of disinfectants on surfaces for virus disinfection, although it has been suggested that a charge of at least 0.1 mC/kg is needed [26]. Four out of the six ESS tested for charge/mass produced sprays above that level.

When switching from DI to tap water, there was a slight increase (~0.01 mC/kg) in the average charge/mass for four of the ESS, although none with evidence indicating statistical significance (p-values ranging from 0.06–0.21). Two of the ESS showed a slight decrease in charge/mass when switching from DI to tap, with the Total 360 model results considered significant (p-value = 0.04). Our somewhat mixed results are consistent with the literature: Maski et al. [10] showed that charge/mass when electrostatically spraying surface water vs. groundwater depended on flow rate. Further, Mamidi et al. [27] showed that charge/mass could increase or decrease with increasing liquid conductivity, and that charge/mass varied approximately only 0.05 mC/kg with a three decade change in conductivity.

The average electrostatic charge results for the Total 360 ESS when spraying the three disinfectants ranged between approximately -1 to -2 mC/kg and were about 75% lower (statistically significant with p-values ≪ 0.05) than when spraying tap or DI water (approximately 6 mC/kg). We were unable to find any charge/mass data in the literature for ESS used for the application of disinfectants, and none of the devices evaluated in this study reported this information either. However, in one study evaluating parameters affecting ESS charge for agricultural pesticide applications, the authors reported an average charge/mass of 1.03 mC/kg [22], and in a similar study [3] evaluating spray deposition in an agricultural setting, charge/mass ranged from 4.8–8.5 mC/kg. Sasaki et al. reported a charge/mass ratio of 1.38 mC/kg at a 1 meter spray distance, and that charge/mass decreased with increasing spray distance [17]. The charge/mass ratios reported in these agricultural-application studies are comparable to the charge/mass ratios of the devices in the present study that utilize alternating current.

### Spray deposition results

The spray deposition results were documented via photographs taken under black light (ultraviolet A) exposure. Example photographs are presented here in the main body of the manuscript; please refer to the S2 File for additional photographs for all the sprayers’ deposition results. These photographs are meant to provide a qualitative, visual understanding of the spray deposition and the “wrap around” effect, or lack thereof.

**Fig 3:**
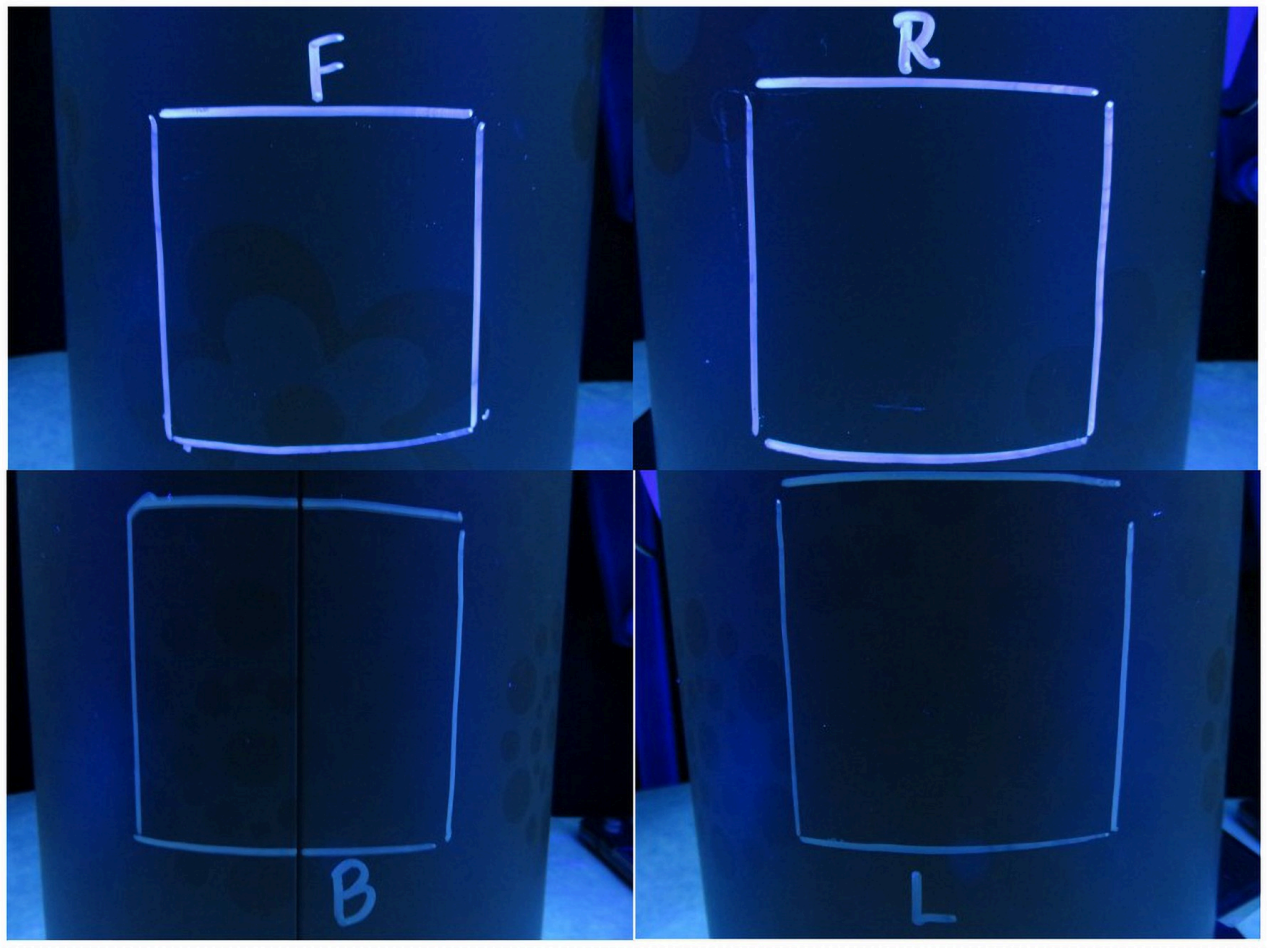
Example photographs for each quadrant of one of the controls (blank, unsprayed) trash cans. F = front of can; R = right side of can; B = back of can; L = left side of can.

Fig 3 is a composite image of four photographs taken of the 3-inch by 3-inch (7.6 cm by 7.6 cm) squares in each quadrant for one of the cylindrical trash cans, prior to spraying the fluorescent dye solution. That is, these are considered blanks or controls, to indicate how the trash cans appeared under black light prior to spraying. As can be seen, all four sides of the can remain relatively obscure, as expected, without the presence of the fluorescent dye solution.

**Fig 4:**
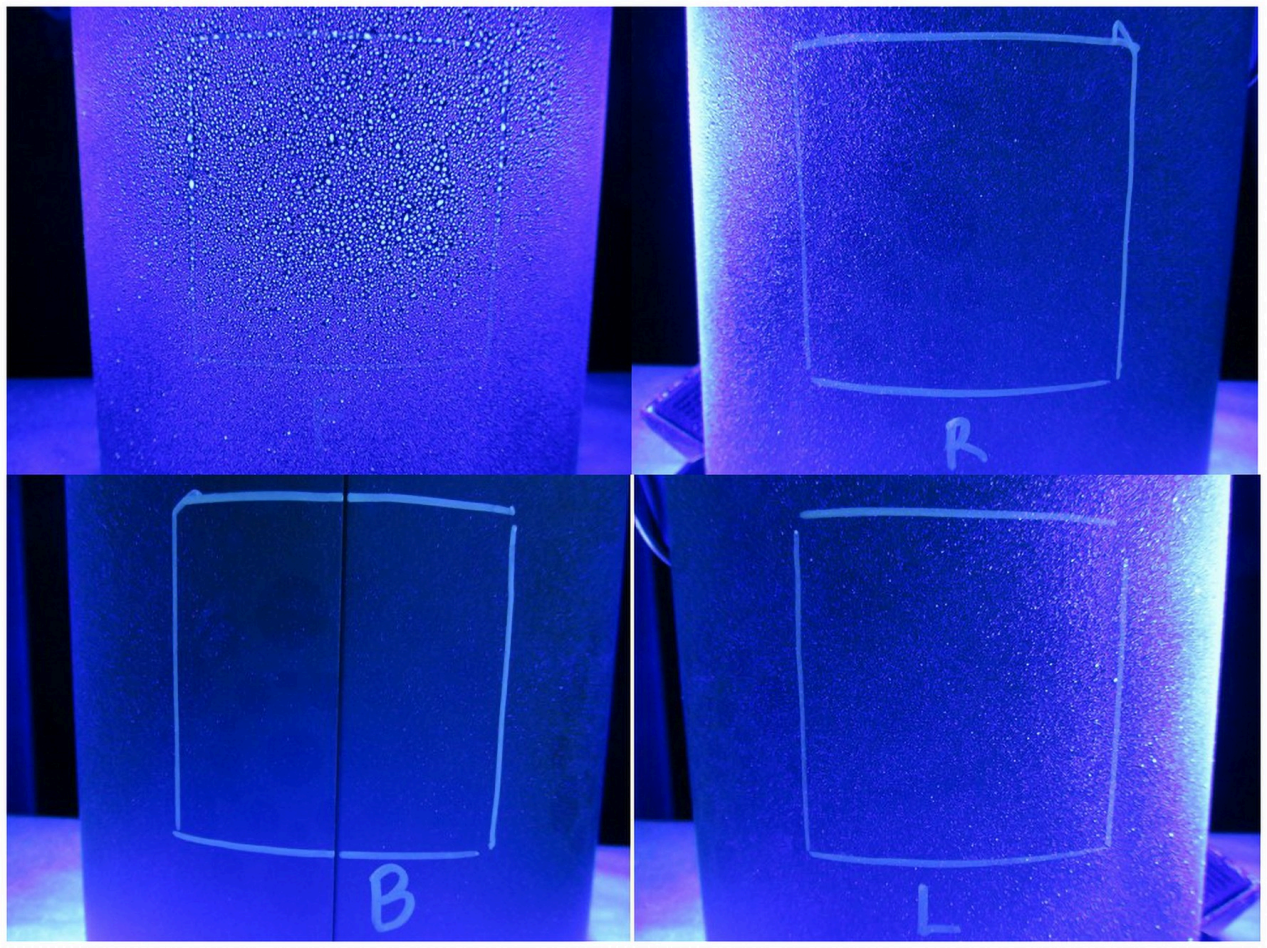
Example photographs for each quadrant of one of the sprayed trash cans. F = front of can (shown in upper left photo); R = right side of can; B = back of can; L = left side of can.

Fig 4 is a composite image similar to Fig 3, but with photo documentation taken after spraying the trash can. The front quadrant of the can shows the most deposition, as expected, with the tiny droplets being visual. In viewing the right quadrant, one can see illumination due to the spray deposition on the front of the can, with some of the spray deposition reaching to about one-third of the square. Results for the left side of the trash can are similar to the right side: only a small amount of deposition occurred within the square, nearer to the front side. The back side of the trash can shows little if any deposition, indicating minimal “wrap-around” effect. Although when compared to the back side of the control trash can (Fig 3), it does not appear to be as obscure.

**Fig 5:**
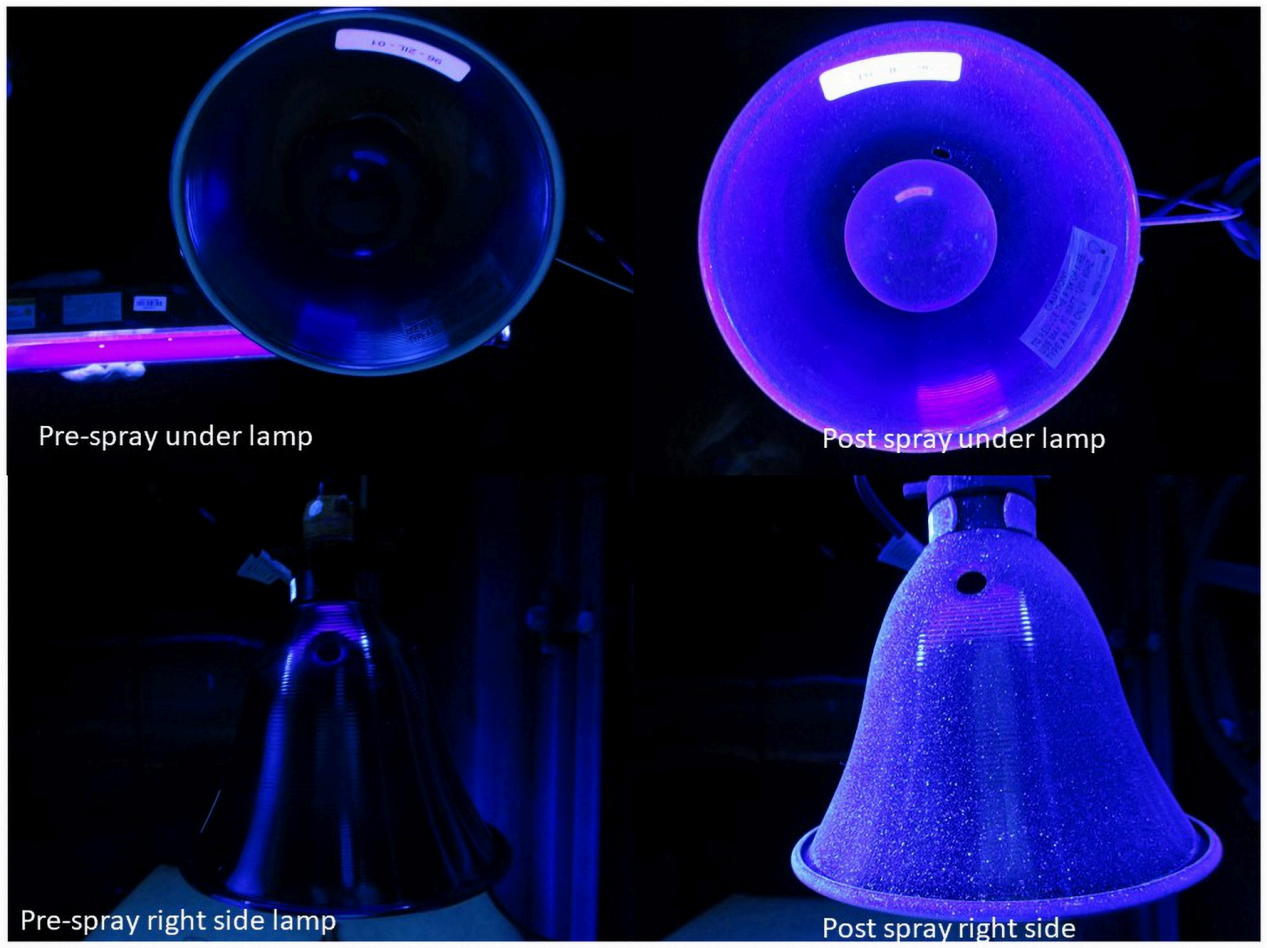
Example photographs of a clip-on lamp, before and after spraying fluorescent dye solution.

Fig 5 is a composite image showing photos of the right side and underside of the small lamp, before and after spraying. The right side of the lamp appears to be well-illuminated after spraying, indicating good coverage and wrap-around, and tiny droplets can be seen. Although the portion of the right-side image near the back of the lamp (opposite of where it was sprayed) does appear somewhat darker, indicating less deposition. There appears to be more deposition on the side of the lamp compared to either side of the trash can, which may be due to the smaller diameter of the lamp. The underside of the lamp also seems to be well illuminated, indicating deposition, although individual droplets are not as visible as they are on the right side of the lamp.

The qualitative deposition results were similar for all the sprayers and foggers evaluated when spraying the trash can, with some minor differences. (Please refer to the S2 File for further details.) That is, as expected, the deposition was the greatest at the front of the can, with some minor amounts of spray deposited on the sides (but with more deposited towards the front), and only minimal amounts deposited on the back of the can. The Airofog device appeared to provide the least deposition, consistent with it not providing an electrostatic charge to the spray (Table 4). From the photo-documentation, it appears that the use of tap water versus deionized water did not have any effect on deposition, consistent with the lack of difference in charge/mass for the two fluids (Table 4). And for the ESS in which the electrostatic function could be turned on and off, there didn’t appear to be any difference in deposition results. This lack of effect on spray deposition as a function of whether the electrostatics were switched on or off is mostly consistent with what Sasaki et al. found [17]. We acknowledge and reiterate that these results are only meant to be qualitative in nature, and that a more quantitative approach (such as gravimetric) to measuring deposition and any “wrap-around” effect should be undertaken in future research.

### Fate of disinfectant active ingredient when spraying

The results for the tests to examine loss of the hydrogen peroxide active ingredient via the spray process are summarized in Table 5. As shown in the table, there was no loss in the disinfectant concentration of hydrogen peroxide (after it was diluted per the label requirements) as measured from the sprayer reservoir, when collected at the nozzle, and when sprayed and then collected 3 feet away; these levels were all 0.19–0.20%. The hydrogen peroxide concentration of the undiluted disinfectant, measured several months after it was obtained, was 5.7–6.0%, in contrast to the label indicating it to be 8%.

**Table 5:** Fate of active ingredient when spraying hydrogen peroxide-based disinfectant.

Quantity	Hydrogen peroxide concentration (%) of disinfectant
Undiluted (as shown on the label)	8
Undiluted, measured ~ 5 months from purchase	6
Undiluted, measured ~ 6 months from purchase	5.7
1:32 dilution (label directions for SARS-CoV-2)–collected from reservoir	0.19 ± 0.0
Diluted per label–collected at nozzle	0.19 ± 0.0
Diluted per label–collected 3 feet (1 m) away	0.20 ± 0.0

During the three spray tests, which took place over the course of 1.5 hours, the average vapor phase concentration of hydrogen peroxide was 0.2 ± 0.05 parts per million (ppm). The highest level of hydrogen peroxide observed in the vapor phase was 0.35 ppm, which lasted approximately 10 seconds and is lower than the Permissible Exposure Limit (8-hr time weighted average) of 1 ppm [28].

The results for the tests to examine the loss of free available chlorine from the dichlor-based disinfectant via the spray process are summarized in Table 6. With the exception of one anomalous datapoint, there was no loss in the disinfectant FAC level as measured from the sprayer reservoir, when collected at the nozzle, and when sprayed and then collected 3 feet away. These levels all ranged from approximately 4,400–5,000 ppm FAC. However, in the first measurement of the sample collected 3 feet away, the FAC was 1,703 ppm and is believed to be an outlier (we believe this is due to unexplained experimental error). The FAC concentration of the prepared disinfectant (4,347 ppm) and as indicated on the label (4,306 ppm) were not significantly different from each other.

**Table 6:** Fate of active ingredient when spraying a dichlor-based disinfectant.

Quantity	Free available chlorine concentration (ppm) of disinfectant
As shown on label (4 tablets per quart)	4,306
As prepared stock solution	4,347
Sampled from reservoir	4,607–5,028
Sampled from nozzle	4,427–4,667
Collected 3 feet (1 m) away	1,703*-4,908

*first reading for 3 ft sample was 1,703 ppm, which is believed to be an outlier since other two samples were both > 4,650 ppm.

During the three spray tests, which took place over the course of 1.5 hours, the average chlorine gas concentration was 0.14 ± 0.02 ppm. The highest level of chlorine gas was 0.19 ppm, which lasted approximately 10 seconds. In contrast, this concentration is lower than the recommended exposure limit (15-minute average) for chlorine gas of 0.5 ppm [29].

Overall, these tests demonstrated that the loss of AI to the air due to spraying the dichlor- and hydrogen peroxide-based disinfectants was minimal (below occupational health levels of concern). Concurrently, the AI concentrations of the liquid disinfectants sprayed and collected 3 feet away from the spray nozzle did not decrease. The minimal loss of AI for the two disinfectants may be due to the relatively low volatility of the AI as well as the short time (< 1 sec) between when the spray is emitted from the device and deposited on the surface.

## Conclusions

The purpose of the study was to evaluate several different sprayers and foggers for parameters related to their use for the application of disinfectants. The following is a summary of some of the findings or conclusions of the study:

- Due to the range in recommended ESS surface coverage, types of surfaces/materials, varying disinfectant chemistries and ESS parameters such as droplet charge, and site-specific environmental conditions, surfaces may not remain wet for the required contact time of the disinfectant.
- Most but not all the devices and spray distances evaluated for droplet size distribution had an average VMD of ≥ 40 microns.
- Four out of the six ESS tested for charge/mass produced sprays of at least 0.1 mC/kg.
- Two out of the six ESS produced sprays carrying a negative charge, while the other four carried a positive charge.
- For all the devices evaluated, there was minimal apparent wrap-around effect of the spray deposition onto an 8-inch diameter cylindrical object, even for the ESS with the highest charge/mass.
- The loss of AI to the air due to spraying the dichlor- and hydrogen peroxide-based disinfectants was minimal (below occupational health levels of concern). Concurrently, the AI concentrations of the liquid disinfectants sprayed and collected 3 feet away from the spray nozzle did not decrease.

## Supporting information

S1 File Cumulative size distributions of the spray for each spray device and fogger evaluated. (PDF)

S2 File Photographic documentation of the spray deposition for each spray device evaluated. (PDF)

S3 File Wetness testing and data. (PDF)
