# Supplementary material for: Evaluation of electrostatic sprayers and foggers for the application of disinfectants in the era of SARS-CoV-2
Source: PLoS One. 2021 Sep 30;16(9):e0257434. doi: 10.1371/journal.pone.0257434 (PMC8483385; doi:10.1371/journal.pone.0257434)
Supplement: S1 File — (PDF) [file pone.0257434.s001.pdf]

Cumulative size distributions of sprayers and foggers

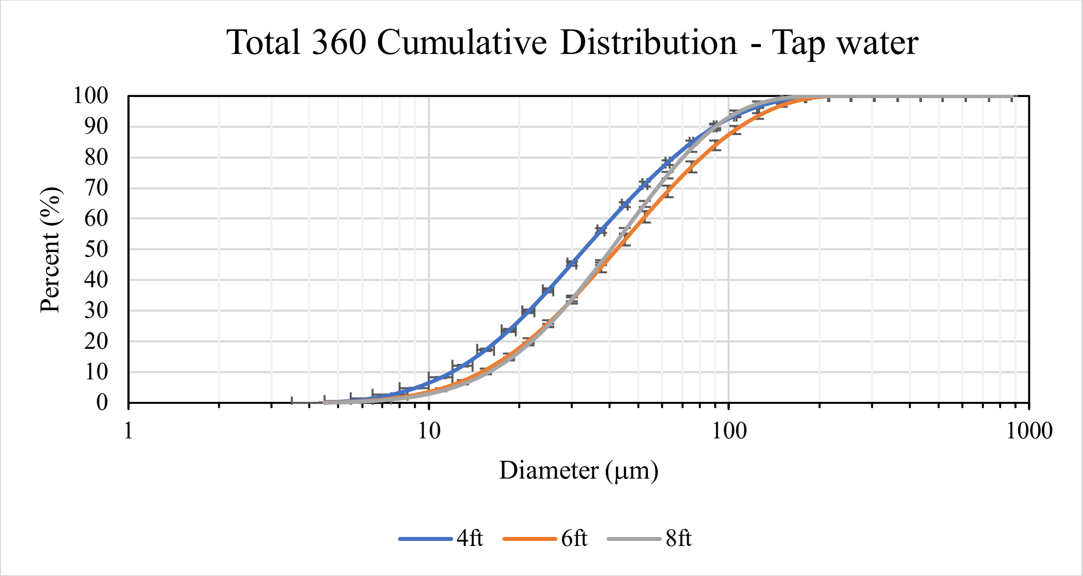

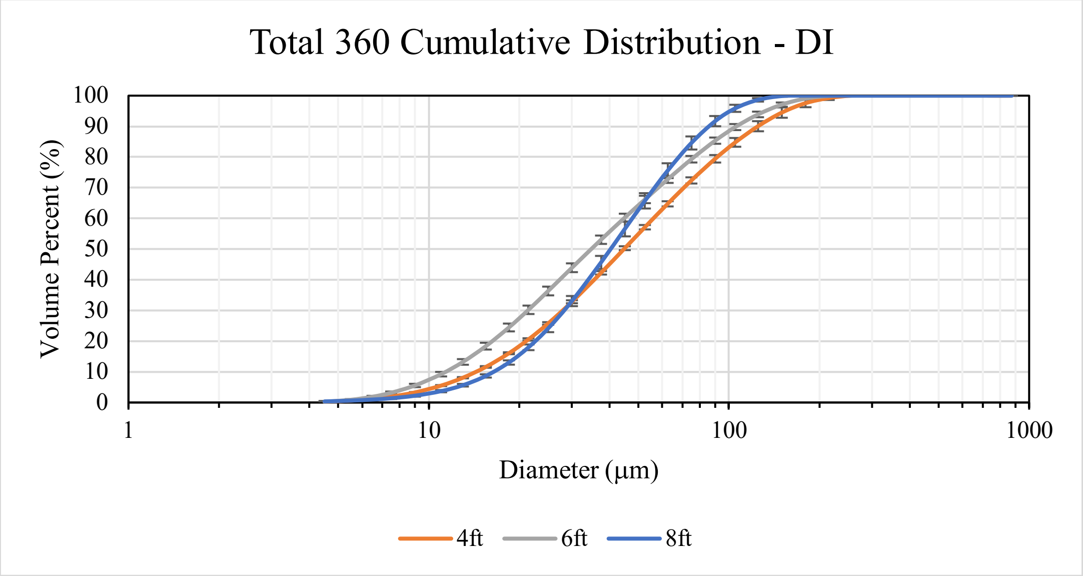

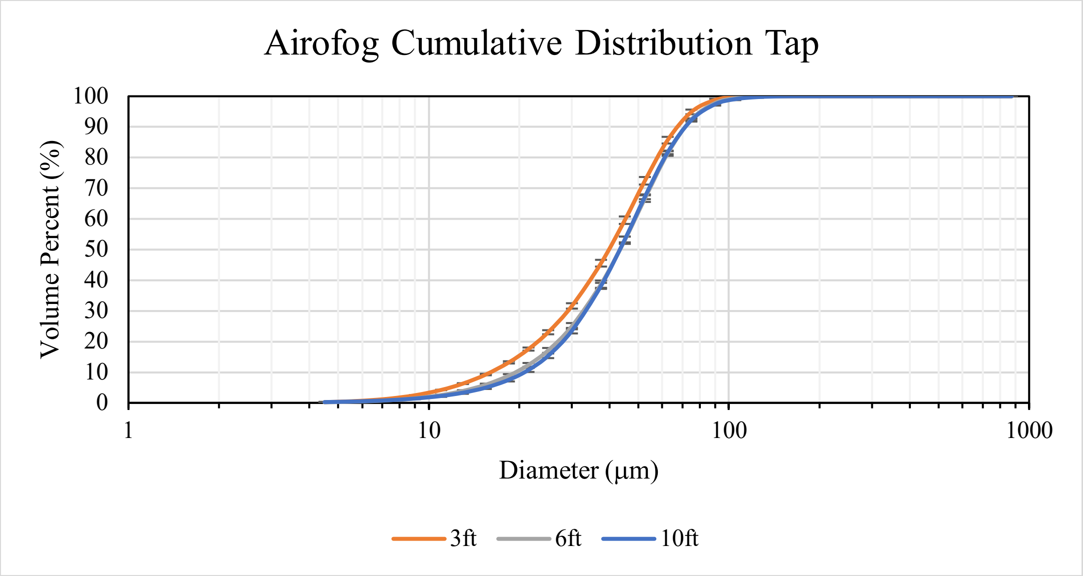
Airofog Cumulative Distribution Tap
Volume Percent (%)
0
10
20
30
40
50
60
70
80
90
100
1
10
100
1000
Diameter (µm)
3ft
6ft
10ft

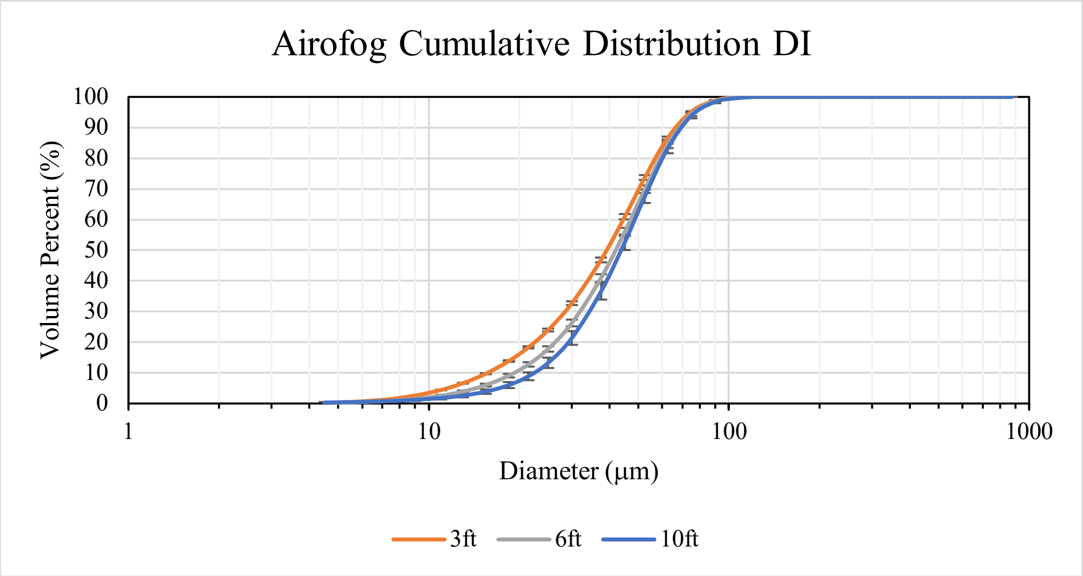
Airofog Cumulative Distribution DI
Volume Percent (%)
0
10
20
30
40
50
60
70
80
90
100
1
10
100
1000
Diameter (µm)
3ft
6ft
10ft

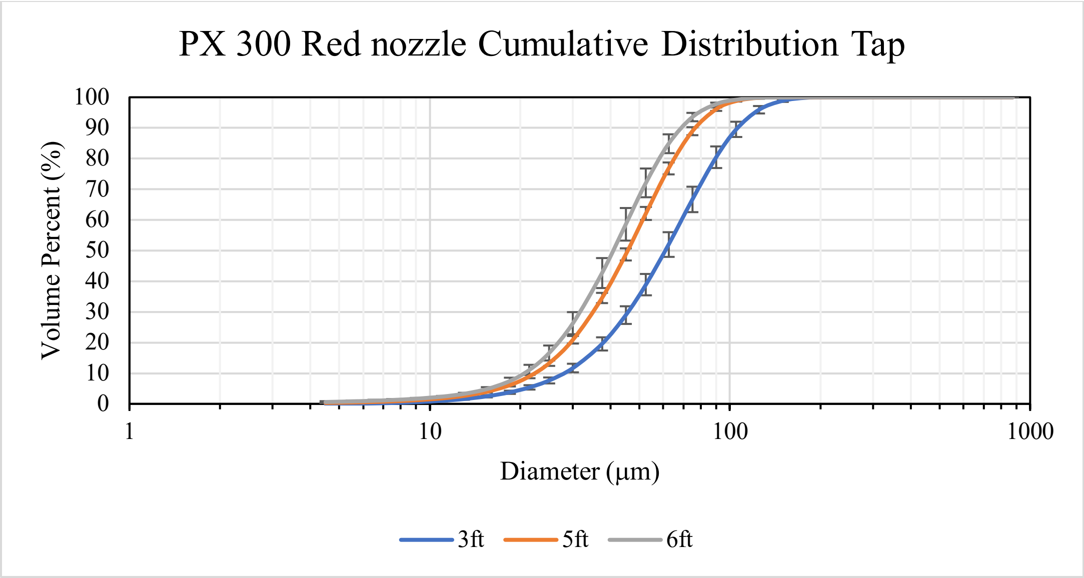
PX 300 Red nozzle Cumulative Distribution Tap
Volume Percent (%)
100
90
80
70
60
50
40
30
20
10
0
1
10
100
1000
Diameter (µm)
3ft
5ft
6ft

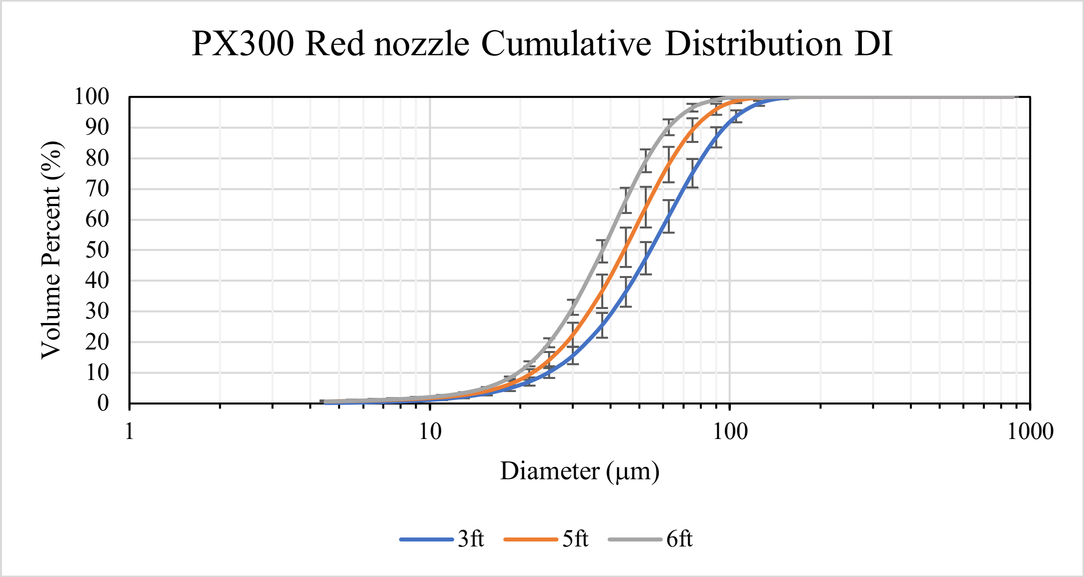
PX300 Red nozzle Cumulative Distribution DI
Volume Percent (%)
100
90
80
70
60
50
40
30
20
10
0
1
10
100
1000
Diameter (µm)
3ft
5ft
6ft

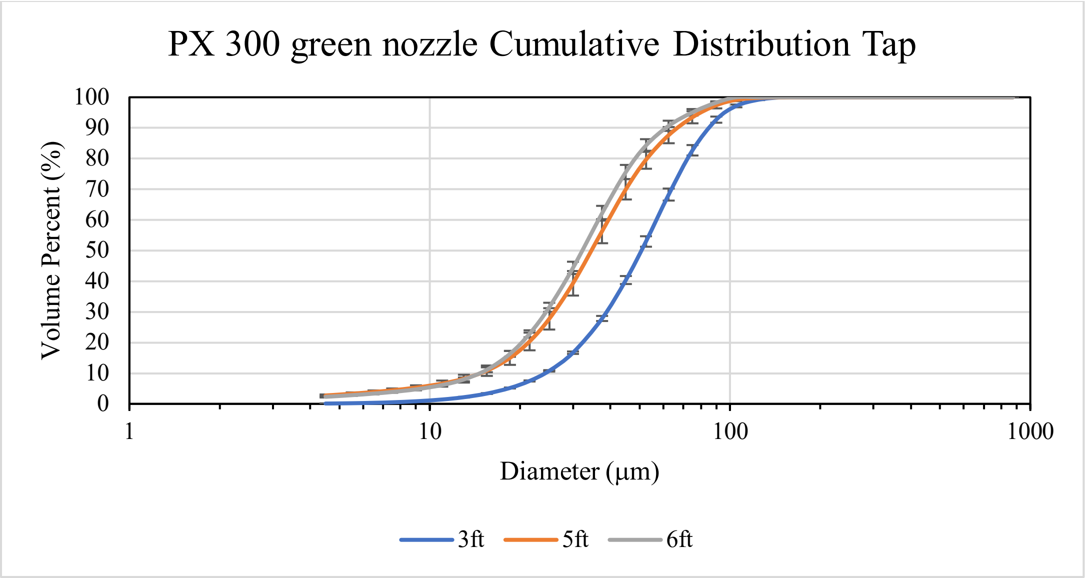
PX 300 green nozzle Cumulative Distribution Tap
Volume Percent (%)
100
90
80
70
60
50
40
30
20
10
0
1
10
100
1000
Diameter (µm)
3ft
5ft
6ft

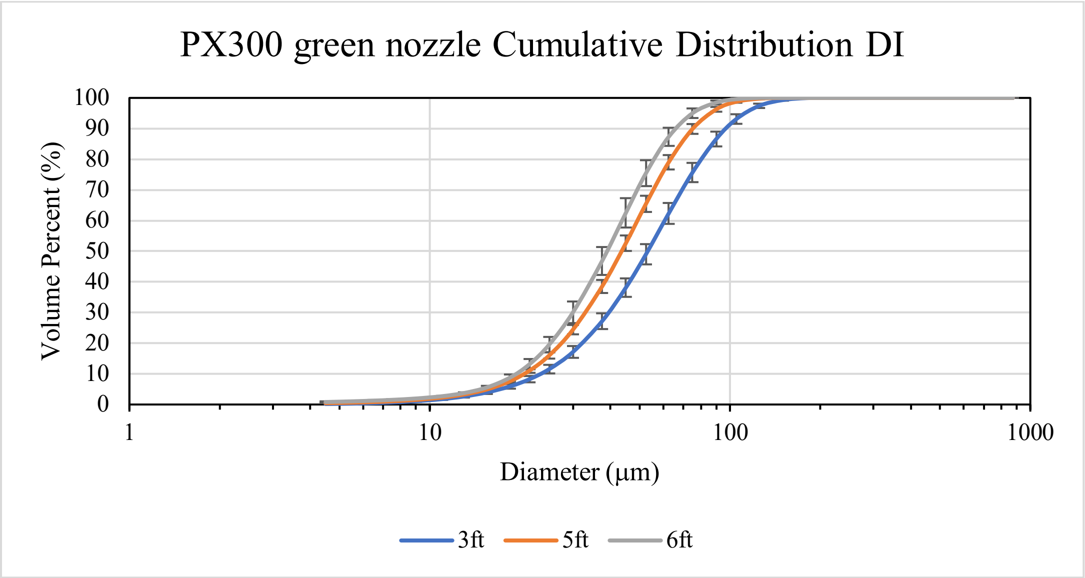
PX300 green nozzle Cumulative Distribution DI
Volume Percent (%)
100
90
80
70
60
50
40
30
20
10
0
1
10
100
1000
Diameter (µm)
3ft
5ft
6ft

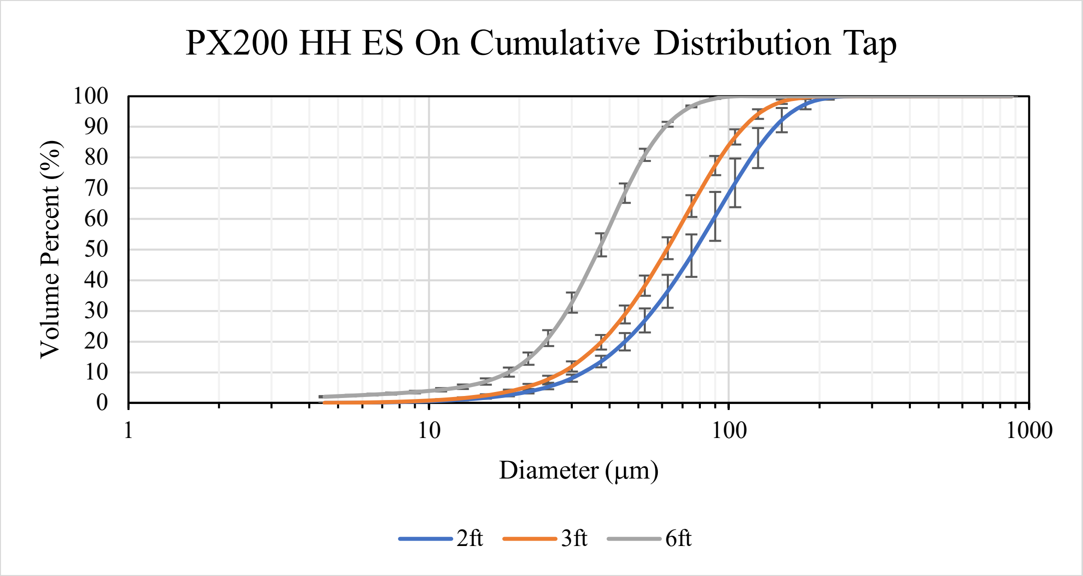
PX200 HH ES On Cumulative Distribution Tap
Volume Percent (%)
Diameter (µm)
0
10
20
30
40
50
60
70
80
90
100
1
10
100
1000
2ft
3ft
6ft

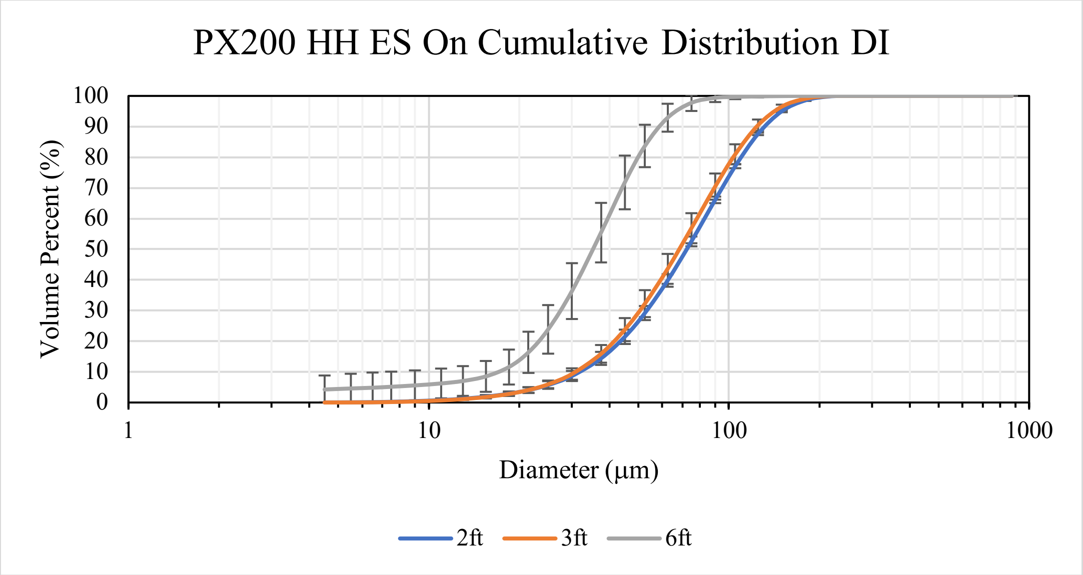
PX200 HH ES On Cumulative Distribution DI
Volume Percent (%)
Diameter (µm)
0
10
20
30
40
50
60
70
80
90
100
1
10
100
1000
2ft
3ft
6ft

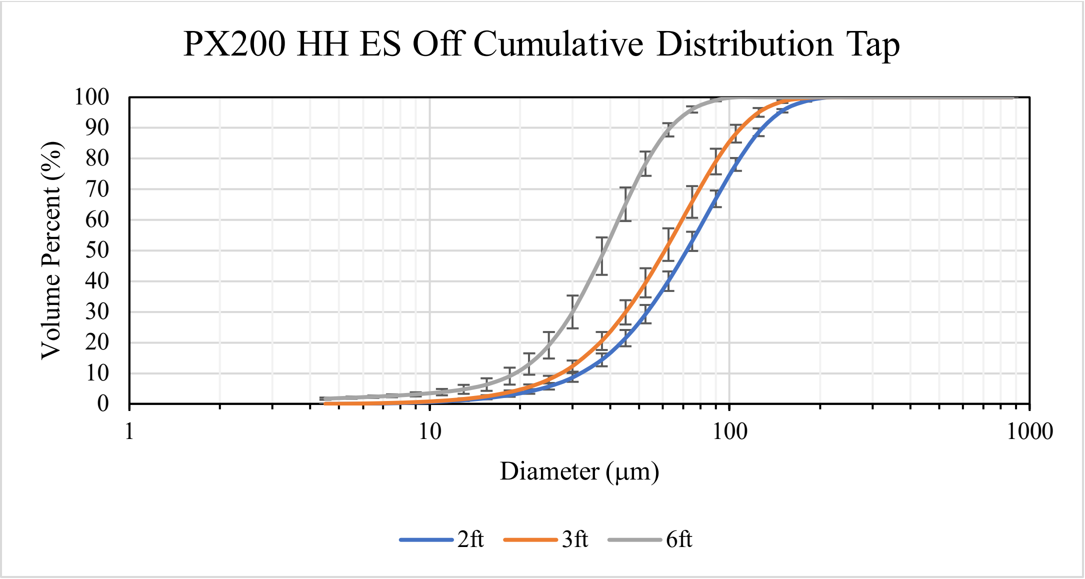
PX200 HH ES Off Cumulative Distribution Tap
Volume Percent (%)
100
90
80
70
60
50
40
30
20
10
0
1
10
100
1000
Diameter (µm)
2ft
3ft
6ft

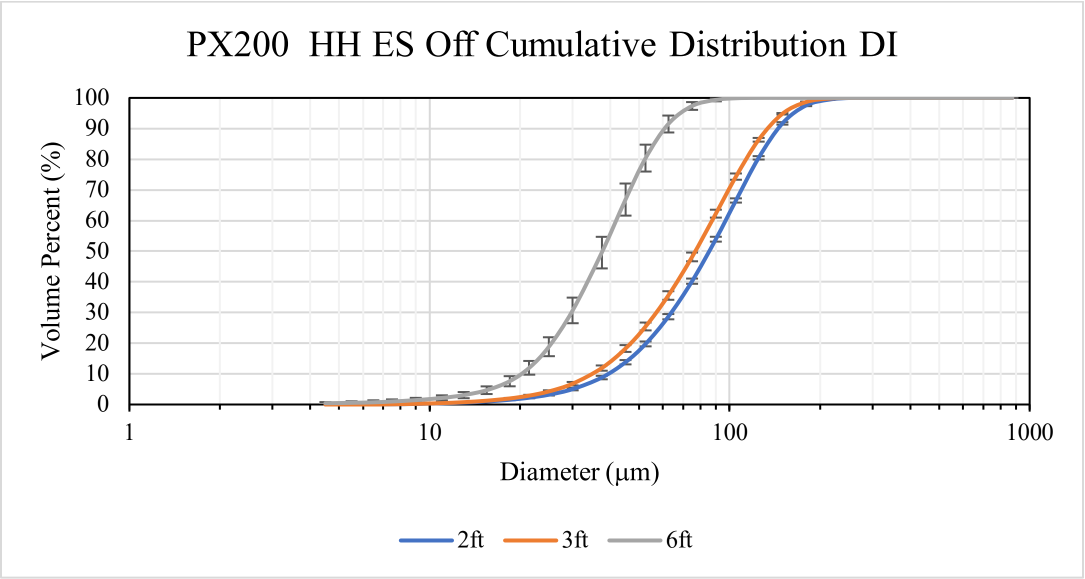
PX200 HH ES Off Cumulative Distribution DI
Volume Percent (%)
100
90
80
70
60
50
40
30
20
10
0
1
10
100
1000
Diameter (µm)
2ft
3ft
6ft

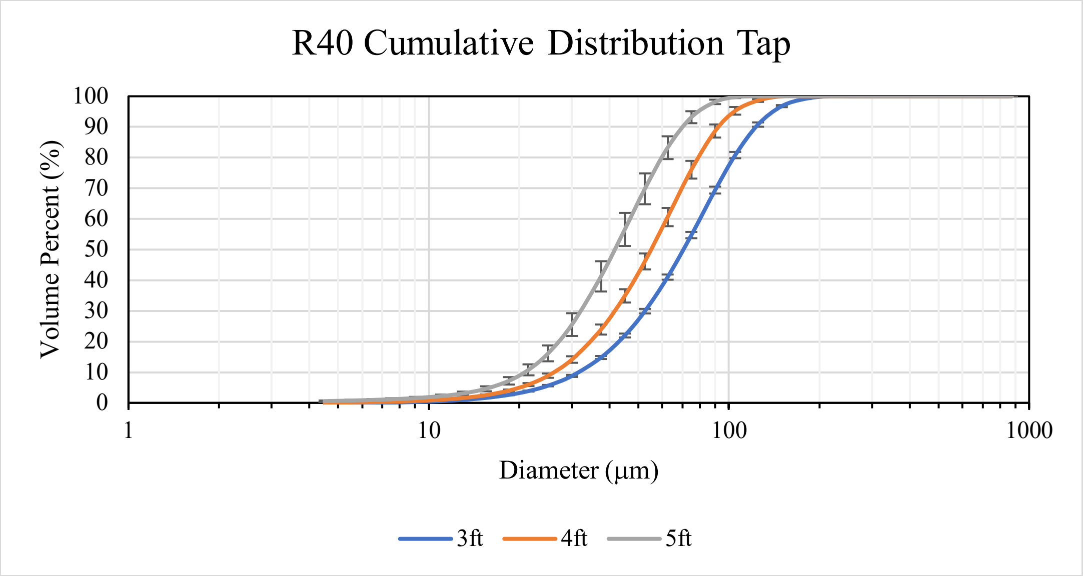
R40 Cumulative Distribution Tap
Volume Percent (%)
100
90
80
70
60
50
40
30
20
10
0
1
10
100
1000
Diameter (µm)
3ft
4ft
5ft

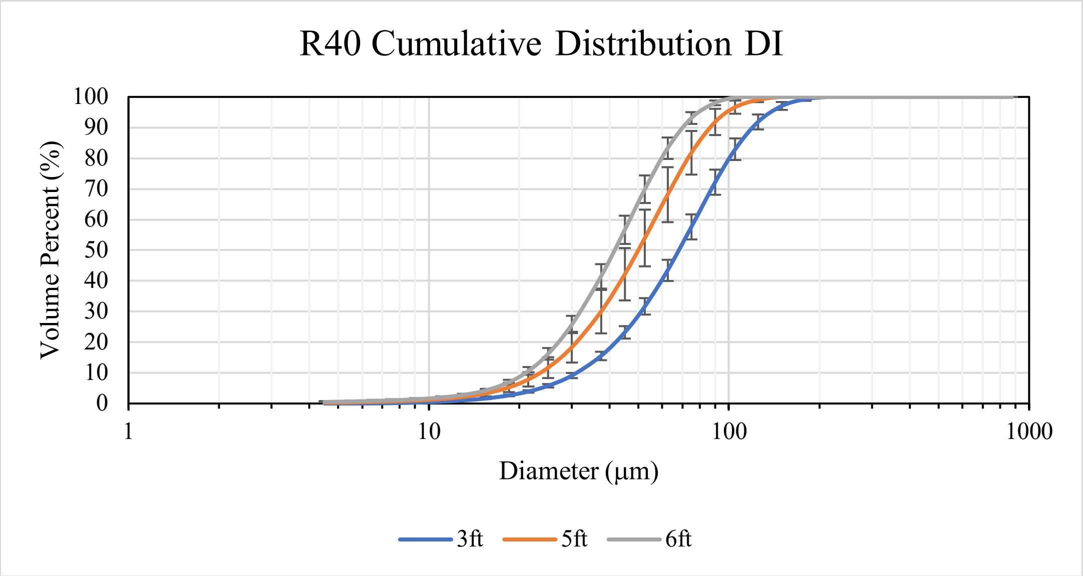
R40 Cumulative Distribution DI
Volume Percent (%)
100
90
80
70
60
50
40
30
20
10
0
1
10
100
1000
Diameter (µm)
3ft
5ft
6ft

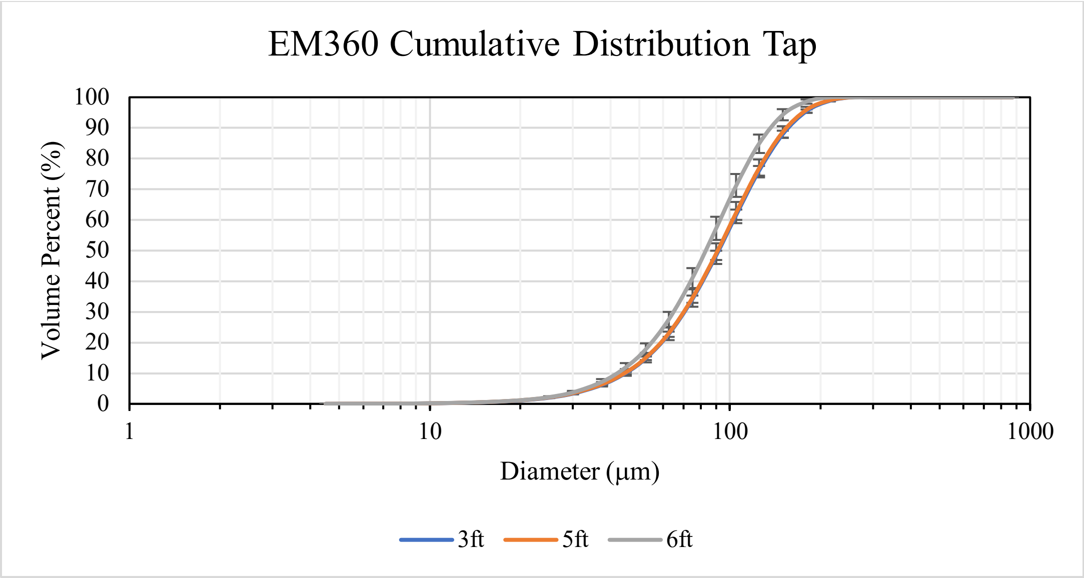
EM360 Cumulative Distribution Tap
Volume Percent (%)
100
90
80
70
60
50
40
30
20
10
0
1
10
100
1000
Diameter (µm)
3ft
5ft
6ft

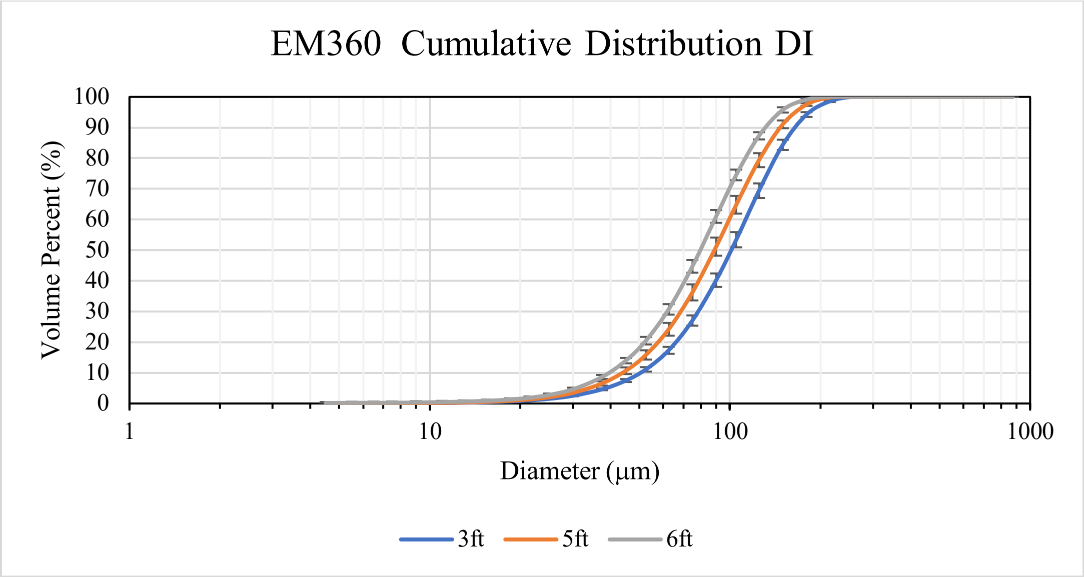
EM360 Cumulative Distribution DI
Volume Percent (%)
100
90
80
70
60
50
40
30
20
10
0
1
10
100
1000
Diameter (µm)
3ft
5ft
6ft

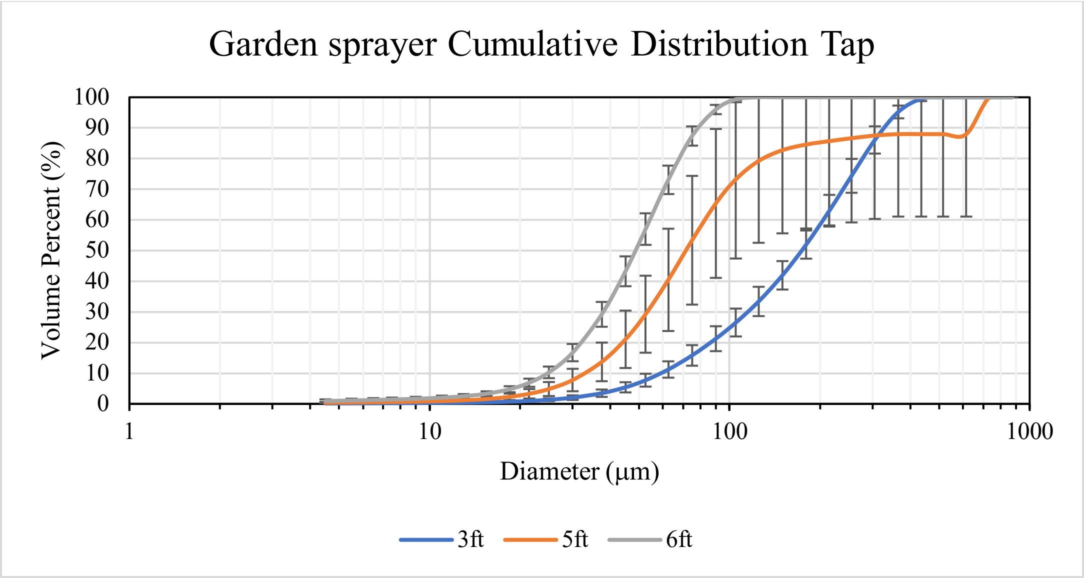

Garden sprayer Cumulative Distribution Tap
Volume Percent (%)
100
90
80
70
60
50
40
30
20
10
0
1
10
100
1000
Diameter (µm)
3ft
5ft
6ft

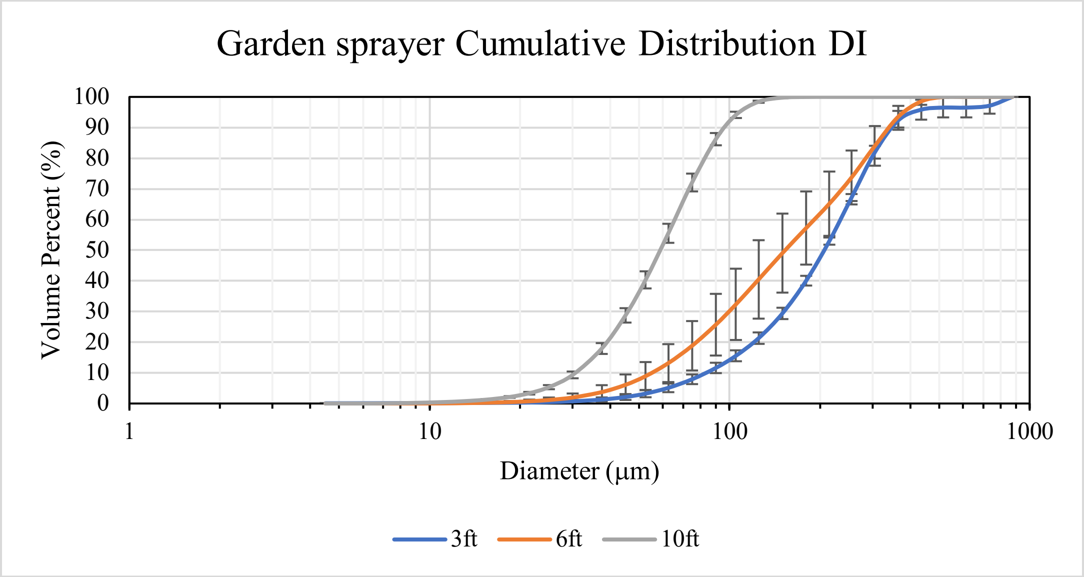

Garden sprayer Cumulative Distribution DI
Volume Percent (%)
100
90
80
70
60
50
40
30
20
10
0
1
10
100
1000
Diameter (µm)
3ft
6ft
10ft

Note cumulative size distribution data not available for the KB-15002E for tap water

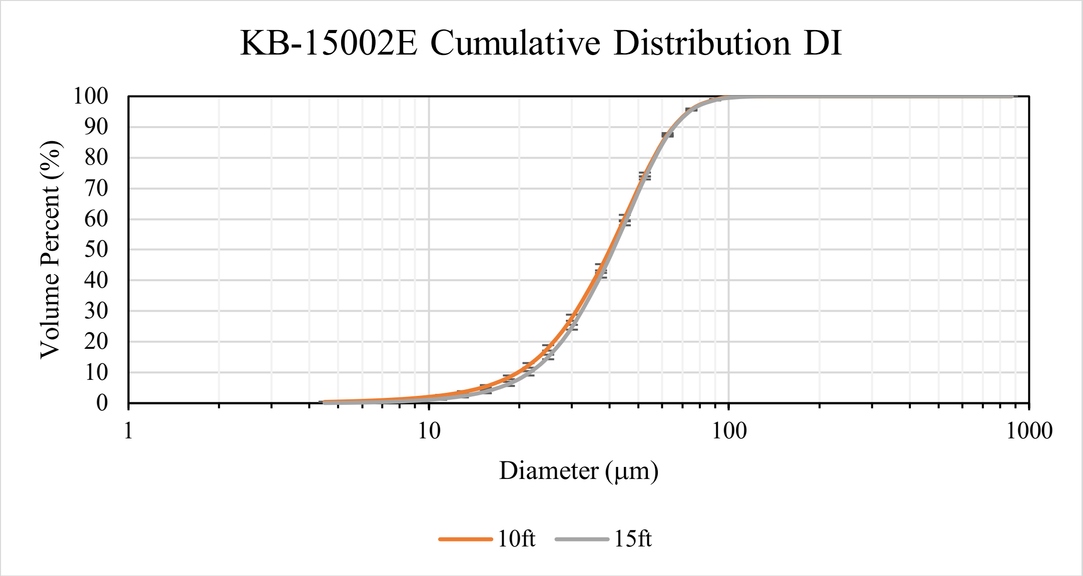

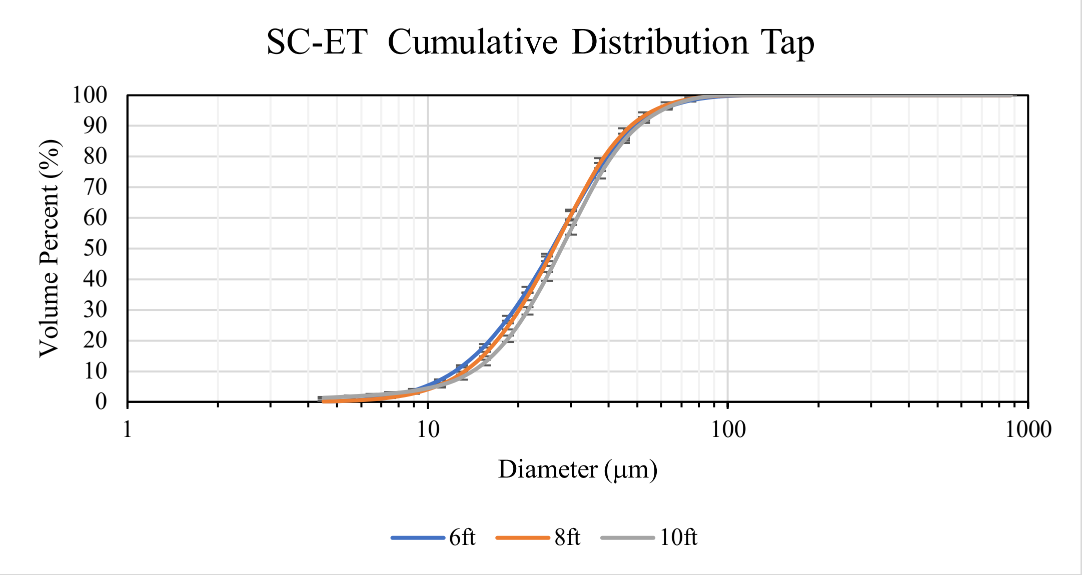
SC-ET Cumulative Distribution Tap
Volume Percent (%)
100
90
80
70
60
50
40
30
20
10
0
1
10
100
1000
Diameter (μm)
6ft
8ft
10ft

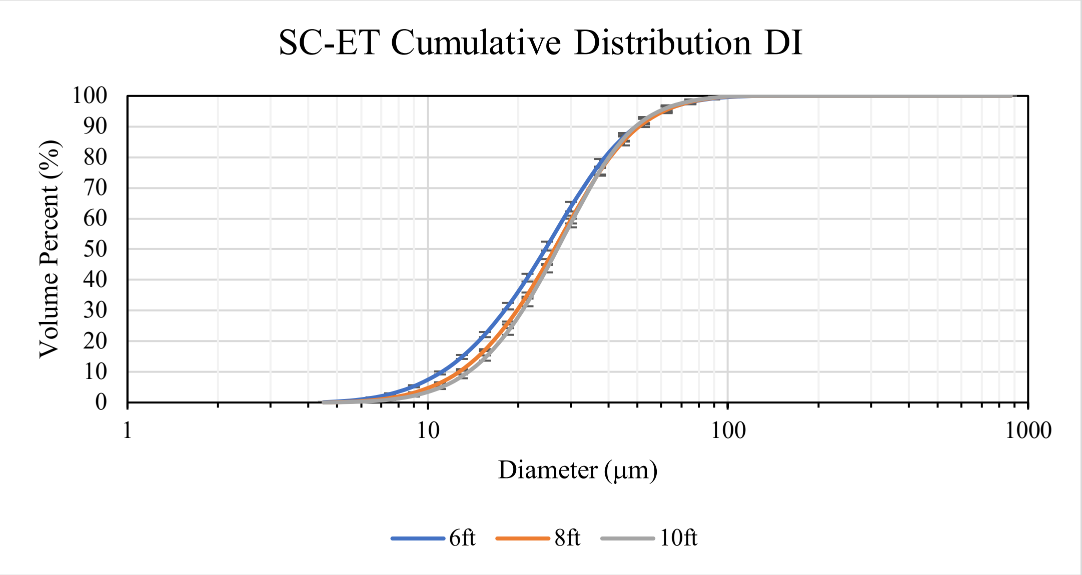
SC-ET Cumulative Distribution DI
Volume Percent (%)
100
90
80
70
60
50
40
30
20
10
0
1
10
100
1000
Diameter (μm)
6ft
8ft
10ft

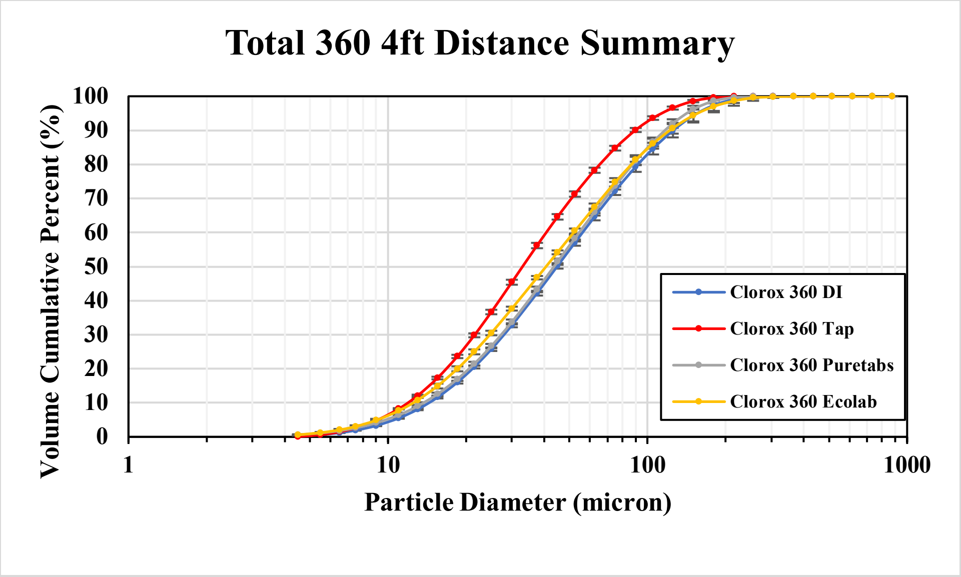
Total 360 4ft Distance Summary
Volume Cumulative Percent (%)
100
90
80
70
60
50
40
30
20
10
0
1
10
100
1000
Particle Diameter (micron)
Clorox 360 DI
Clorox 360 Tap
Clorox 360 Puretabs
Clorox 360 Ecolab

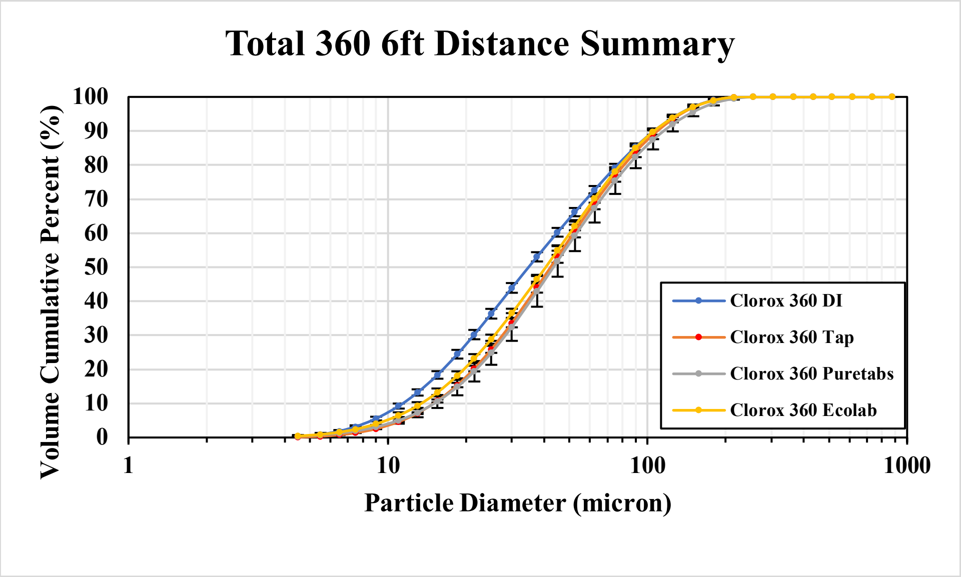
Total 360 6ft Distance Summary
Volume Cumulative Percent (%)
100
90
80
70
60
50
40
30
20
10
0
1
10
100
1000
Particle Diameter (micron)
Clorox 360 DI
Clorox 360 Tap
Clorox 360 Puretabs
Clorox 360 Ecolab

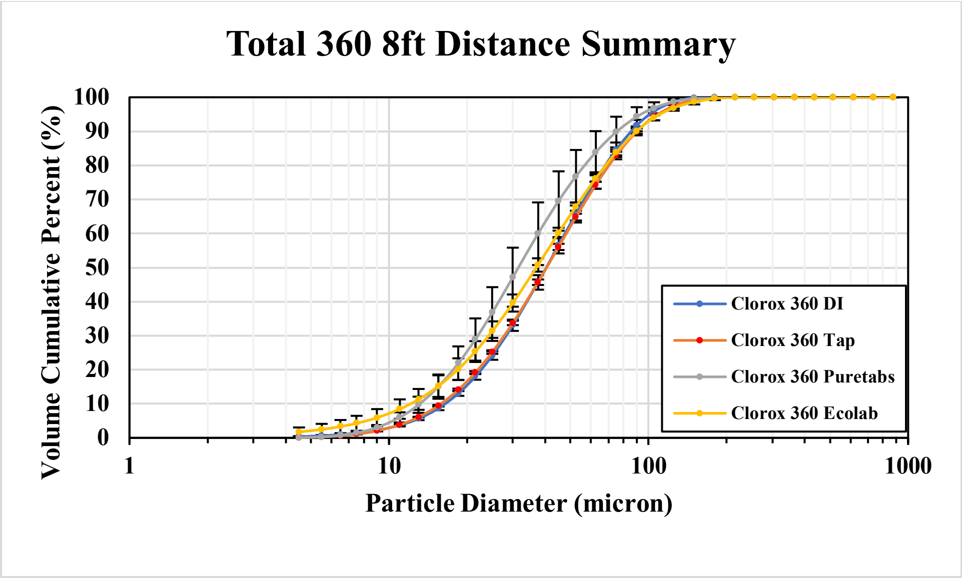

Total 360 8ft Distance Summary
Volume Cumulative Percent (%)
100
90
80
70
60
50
40
30
20
10
0
1
10
100
1000
Particle Diameter (micron)
Clorox 360 DI
Clorox 360 Tap
Clorox 360 Puretabs
Clorox 360 Ecolab
