# Supplementary material for: Evaluation of electrostatic sprayers and foggers for the application of disinfectants in the era of SARS-CoV-2
Source: PLoS One. 2021 Sep 30;16(9):e0257434. doi: 10.1371/journal.pone.0257434 (PMC8483385; doi:10.1371/journal.pone.0257434)
Supplement: S2 File — (PDF) [file pone.0257434.s002.pdf]

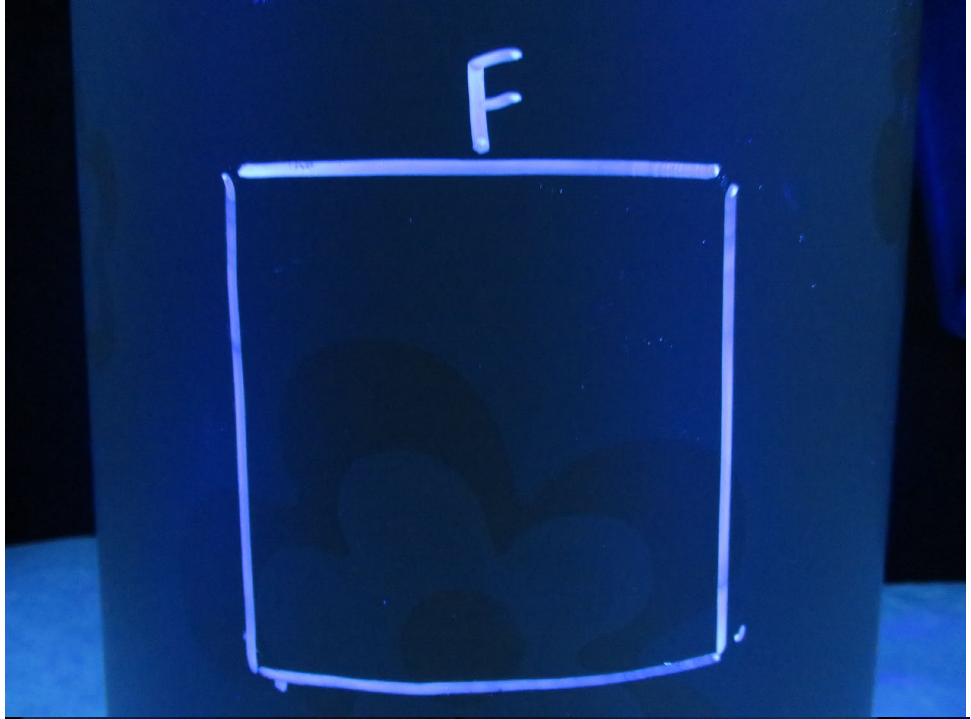


blanks

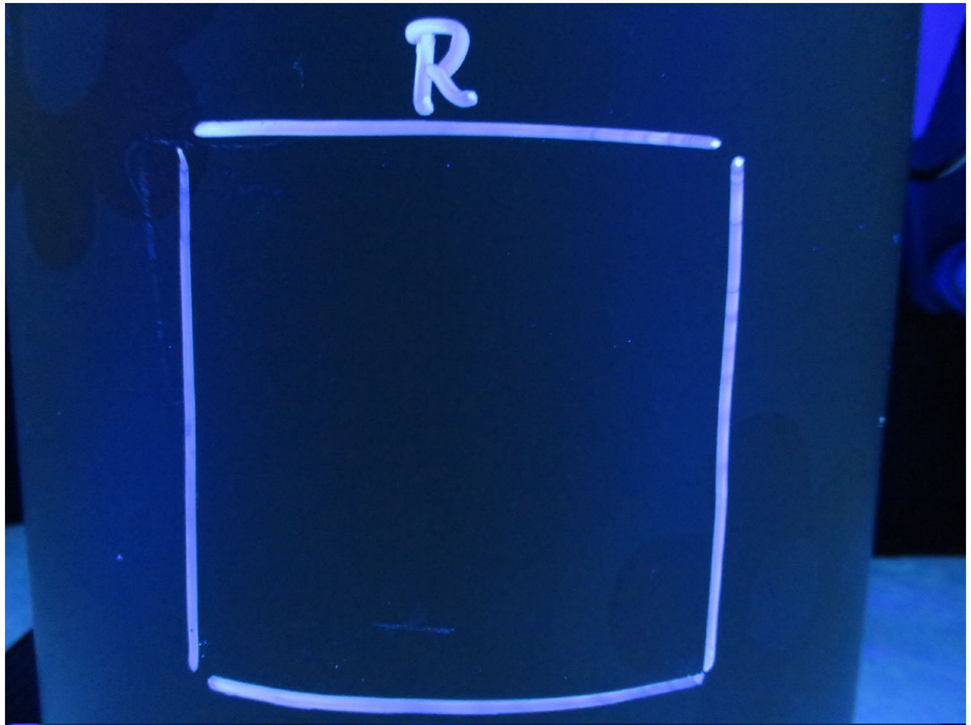


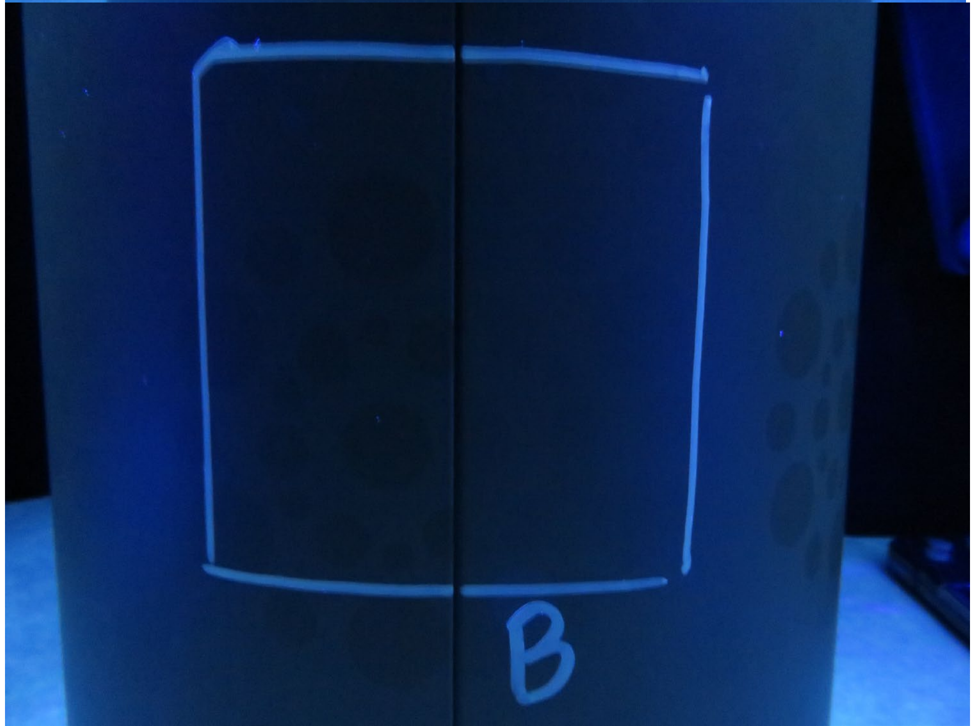


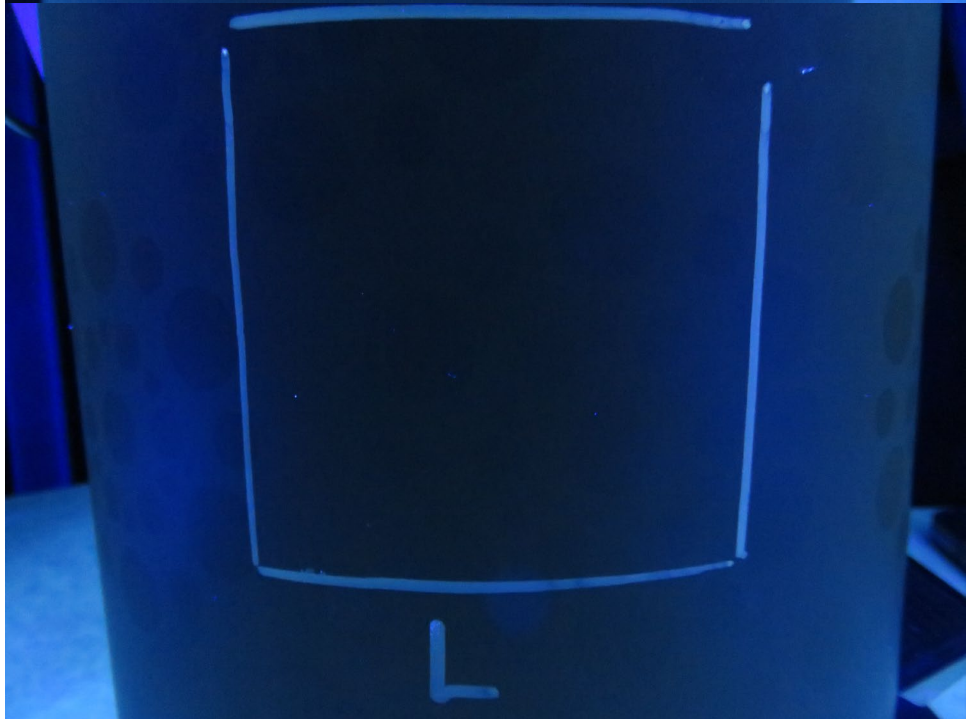

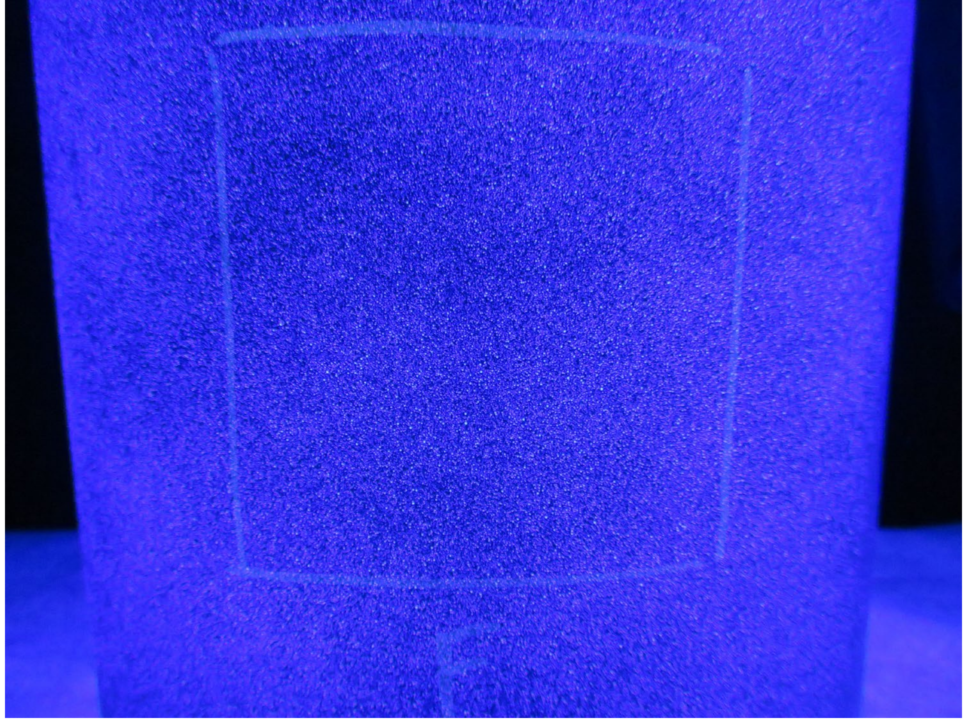

PX300 red nozzle

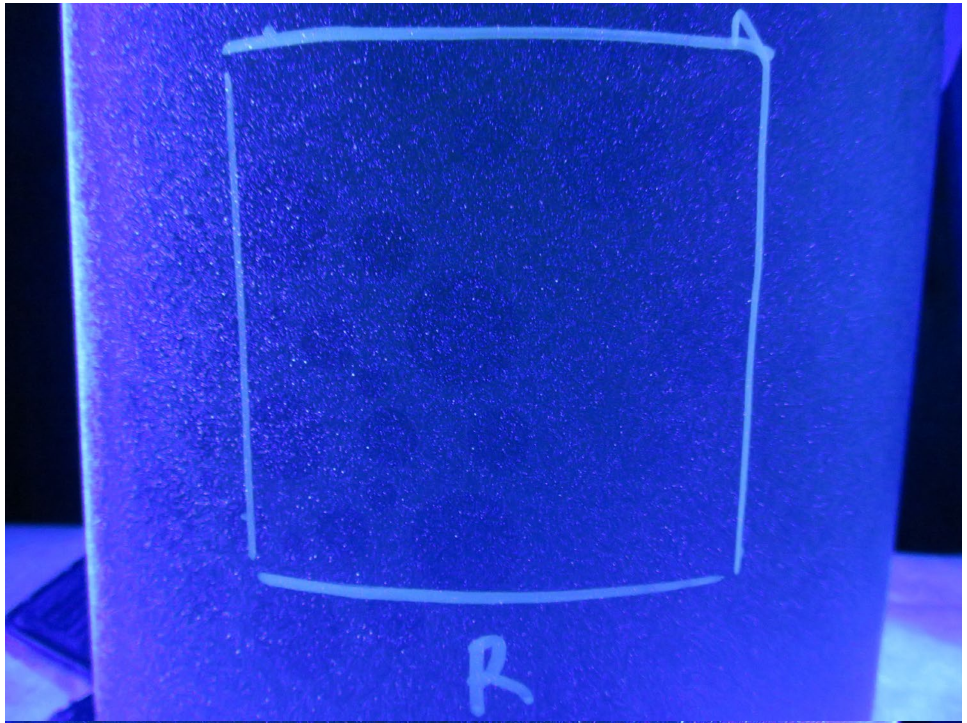


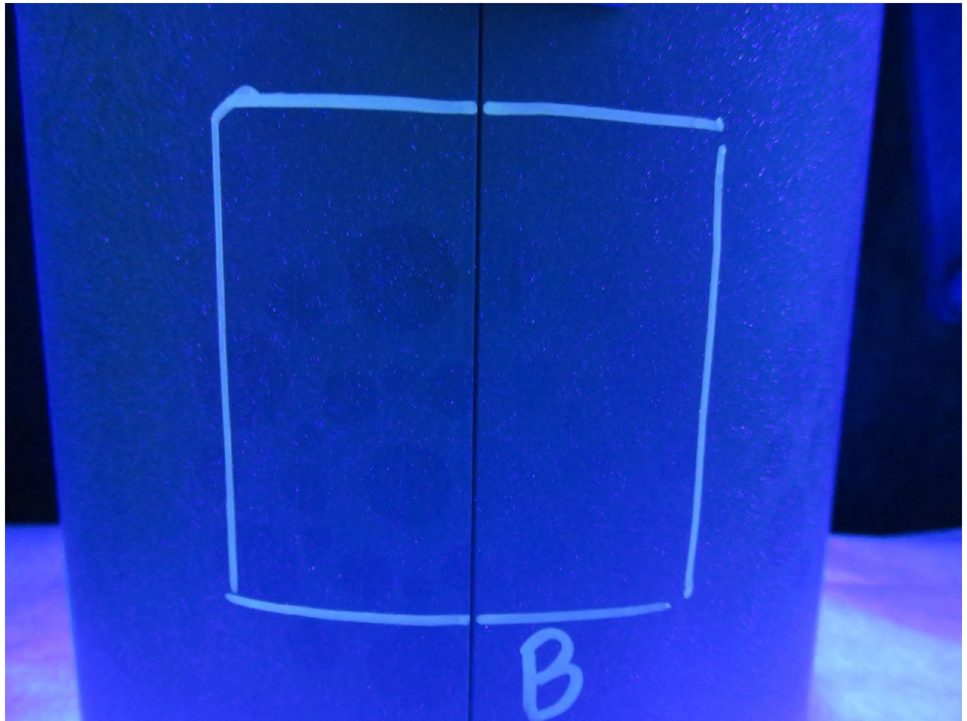


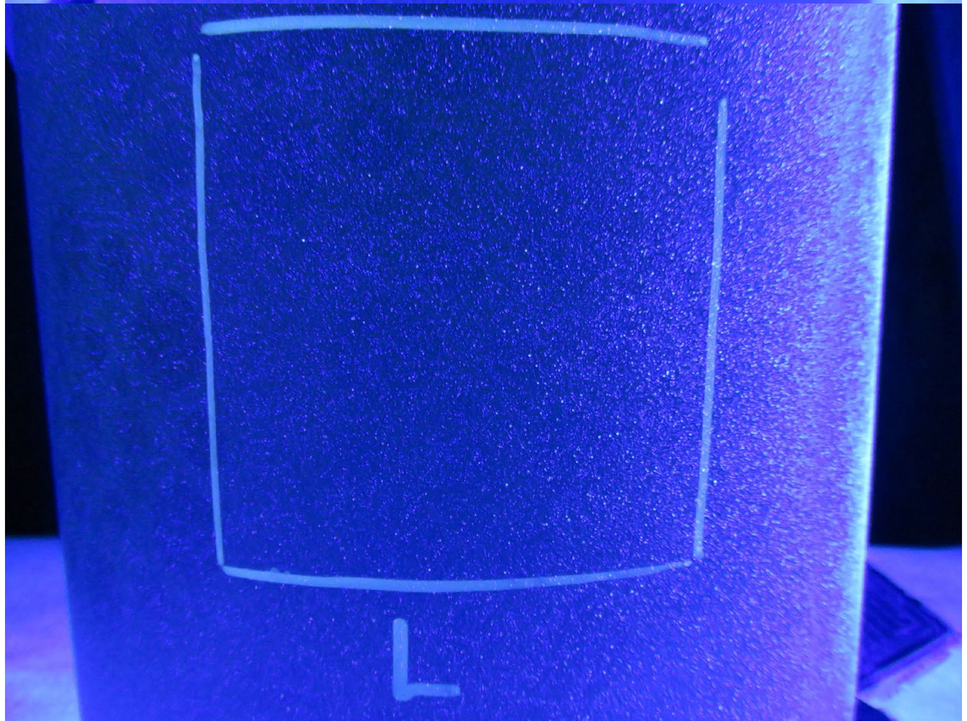

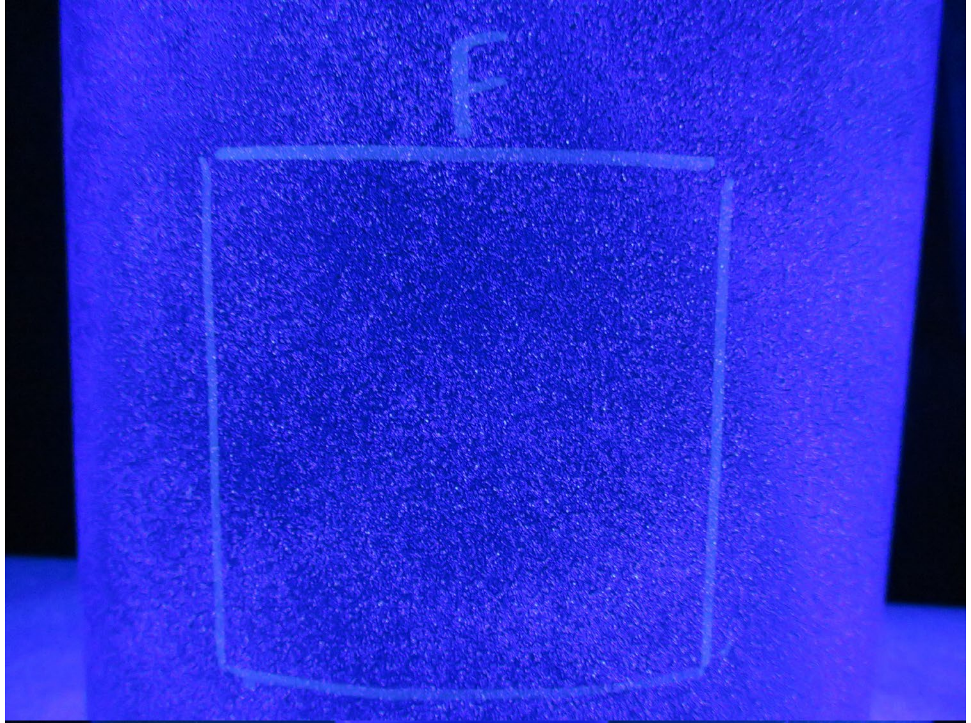


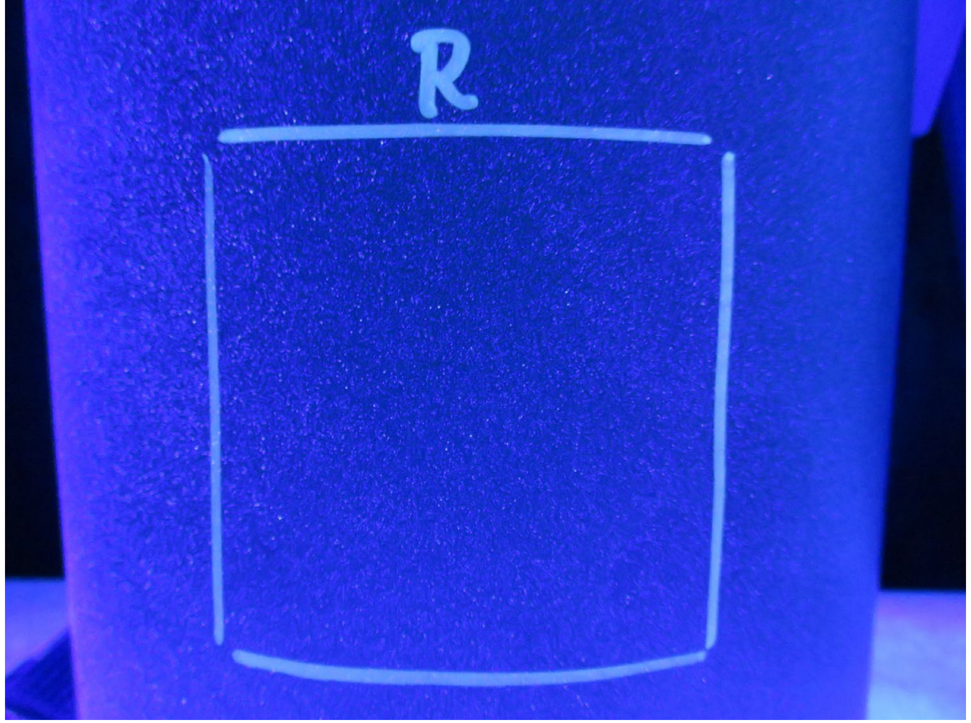


Px300 green

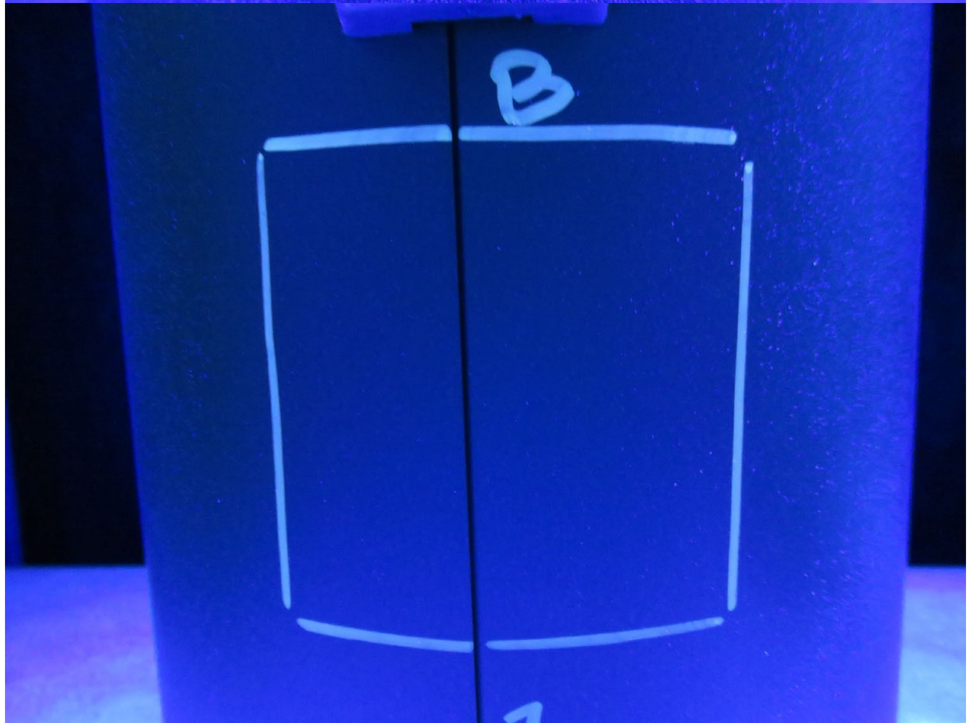


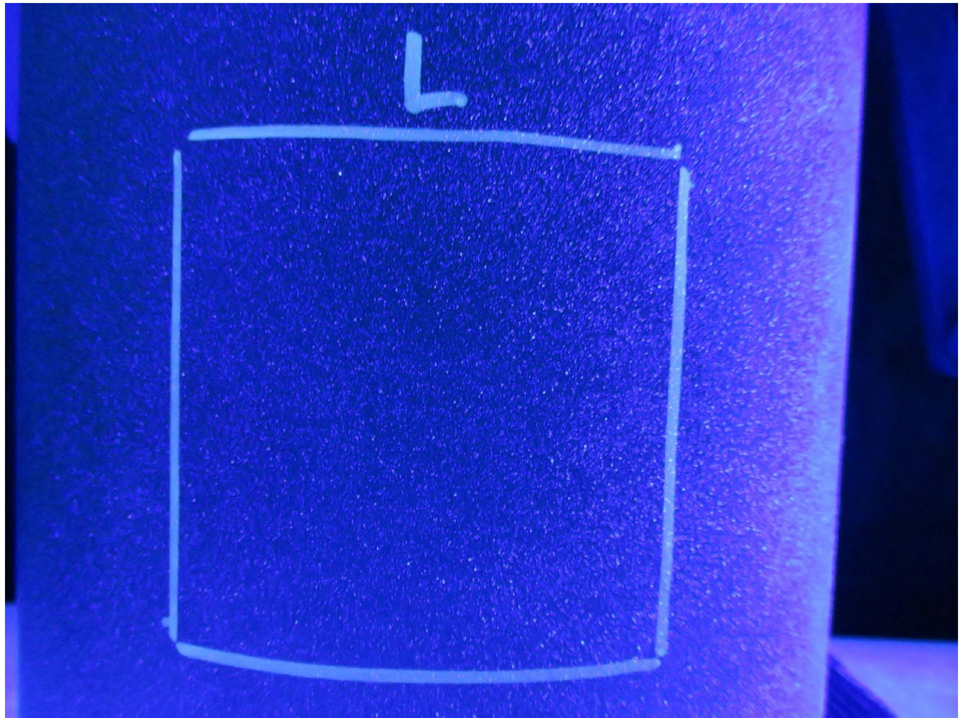

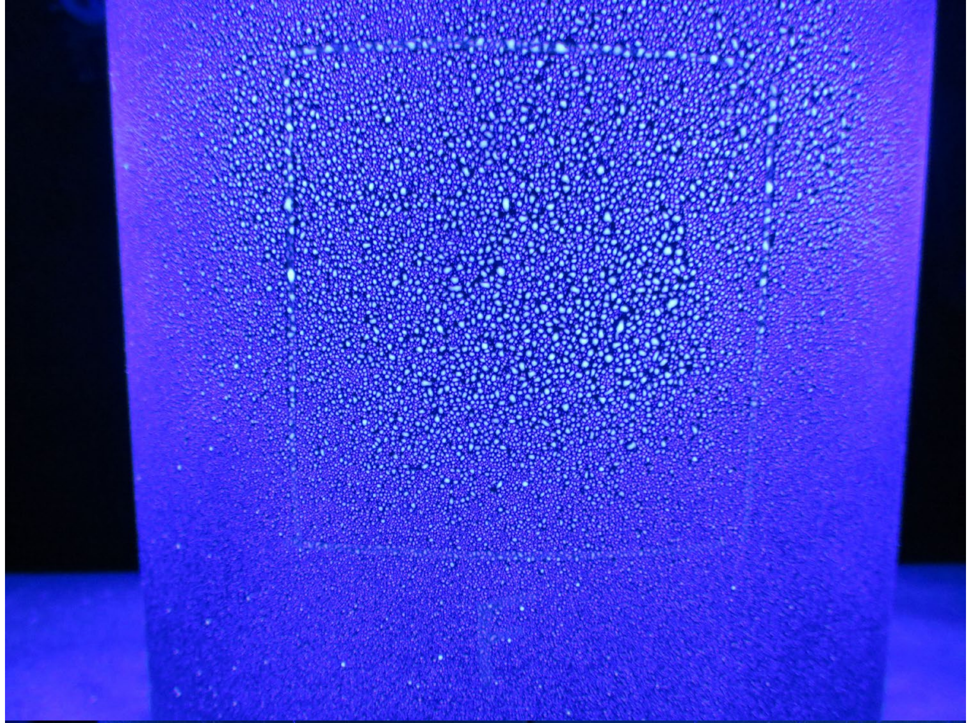

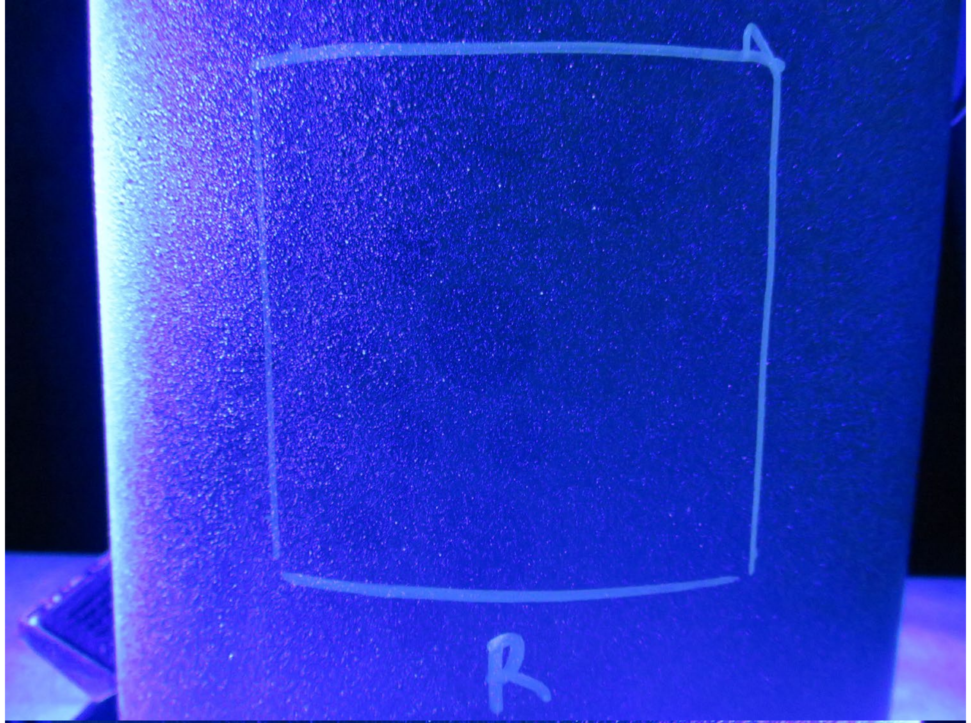


Px200 on

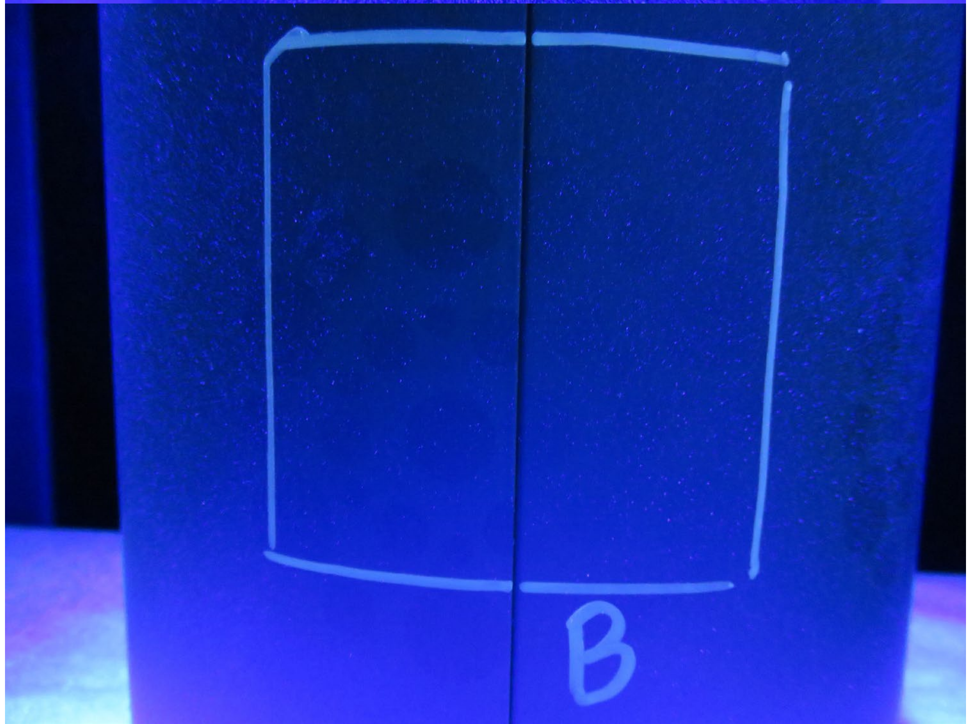


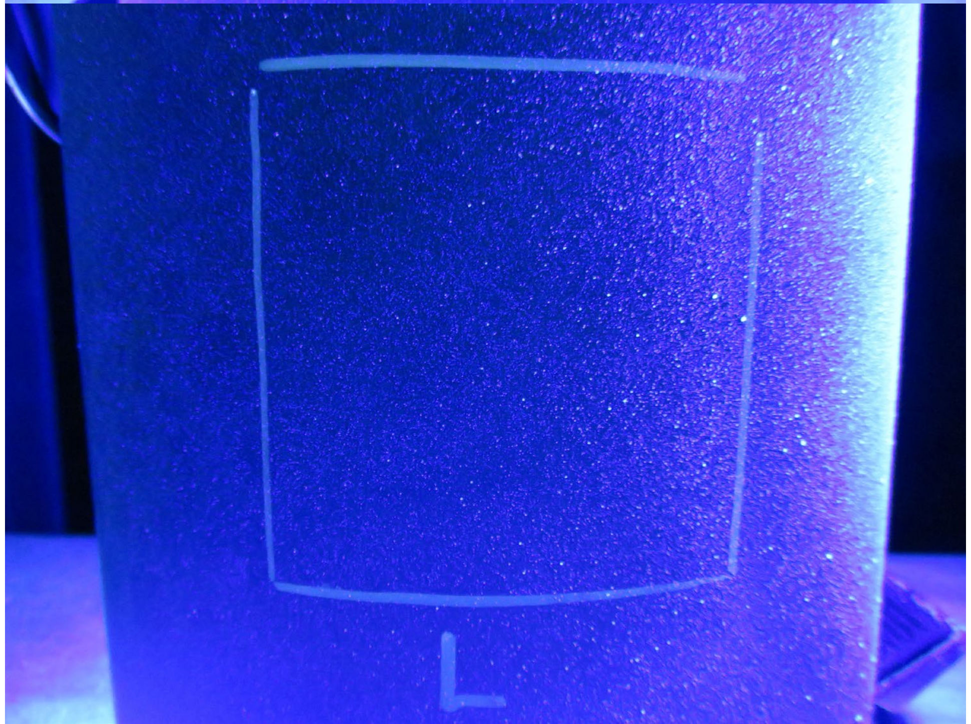

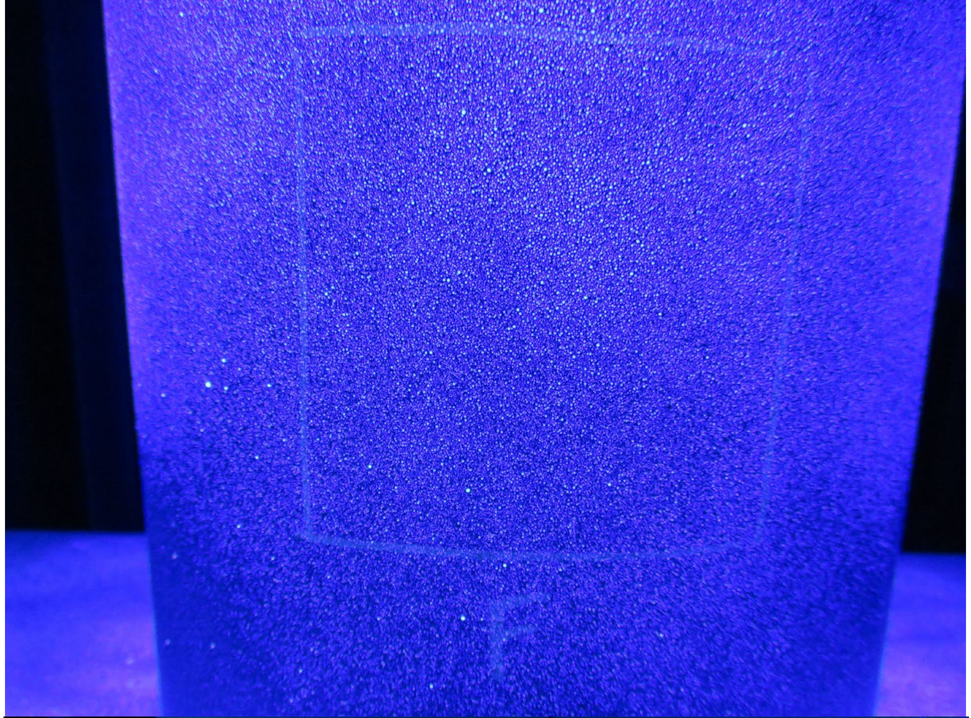


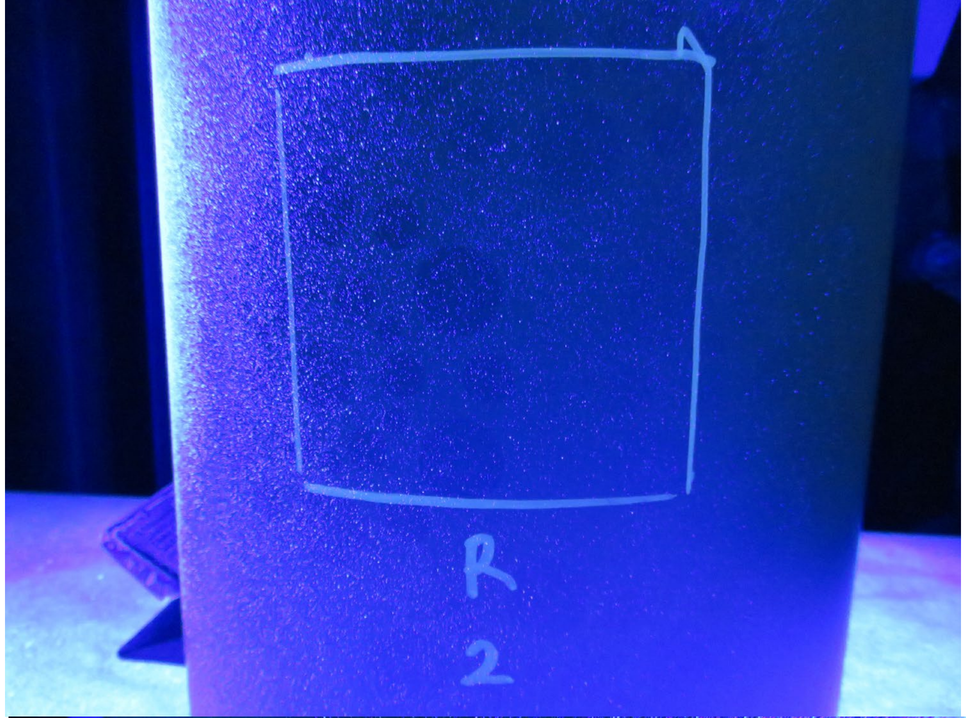


Px200 off

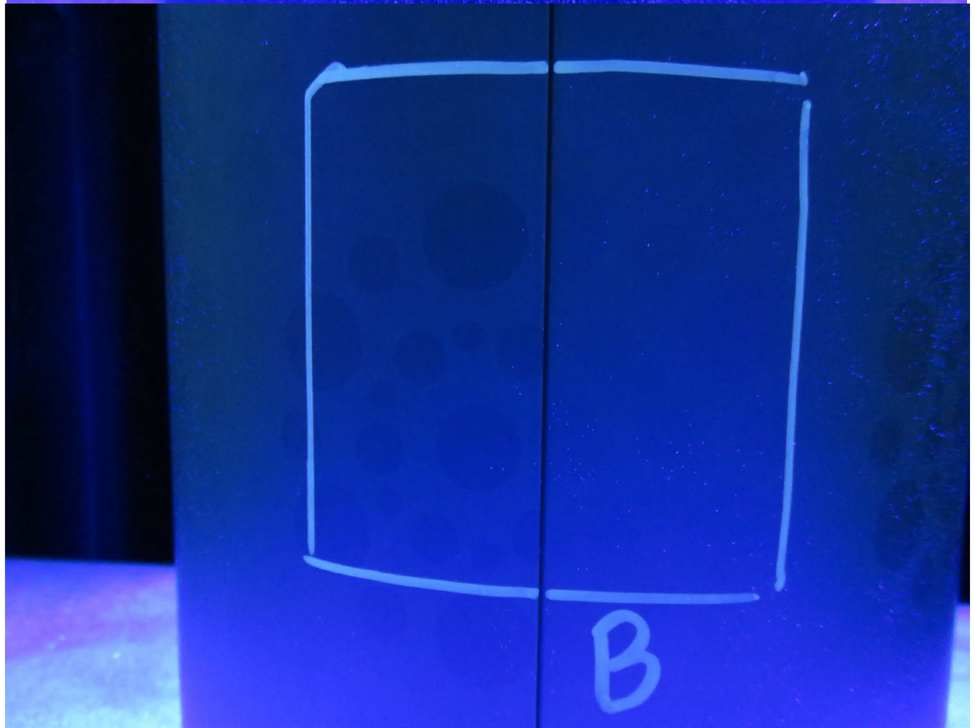


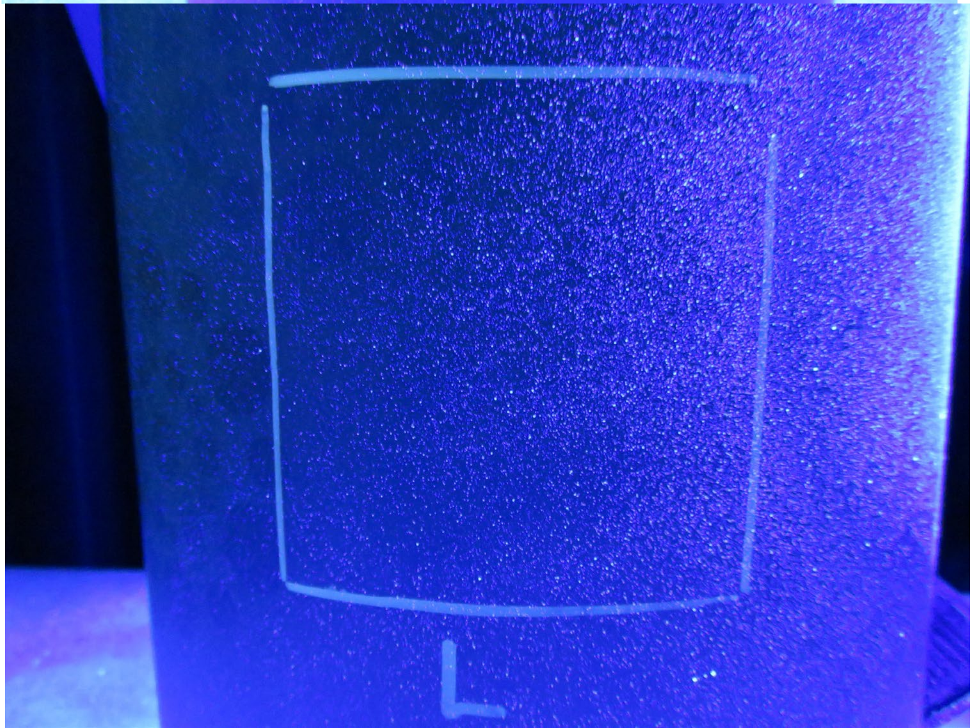

Clorox 360 tap

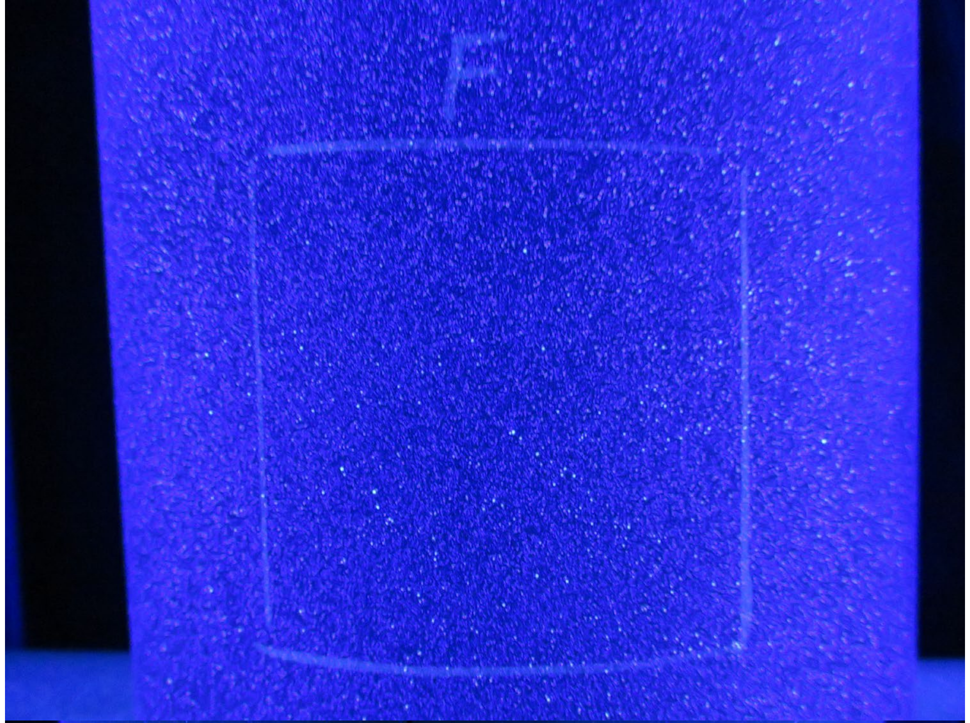


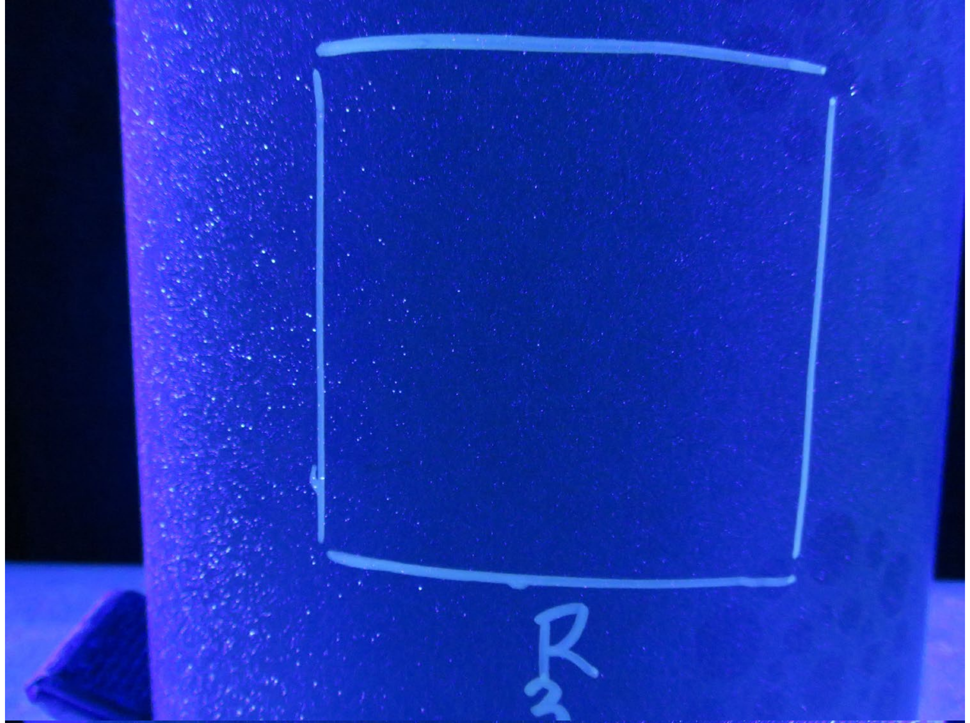


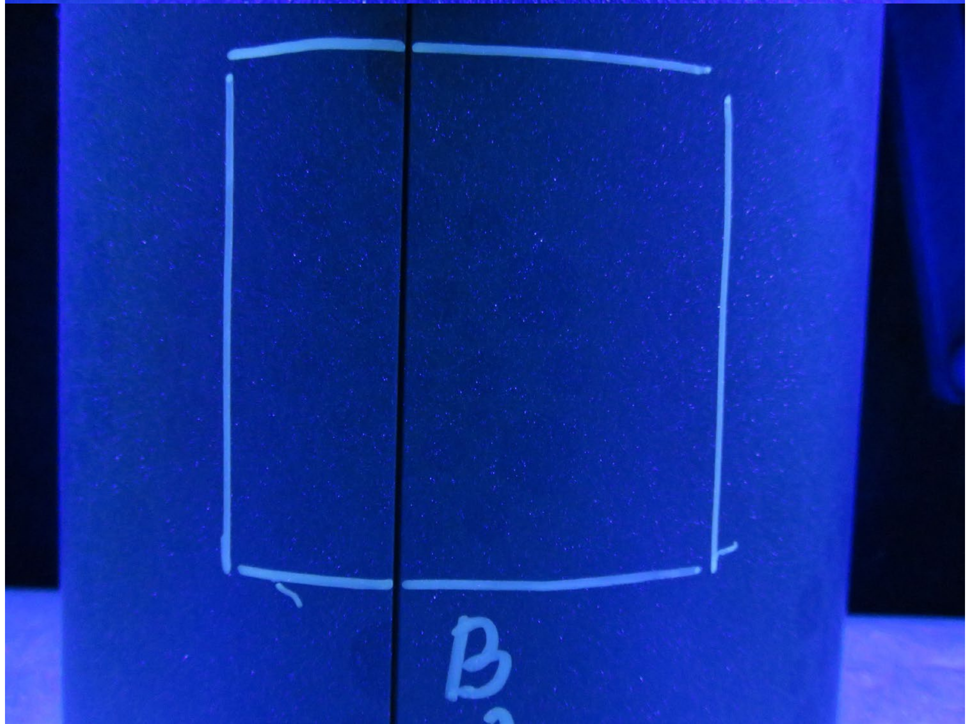


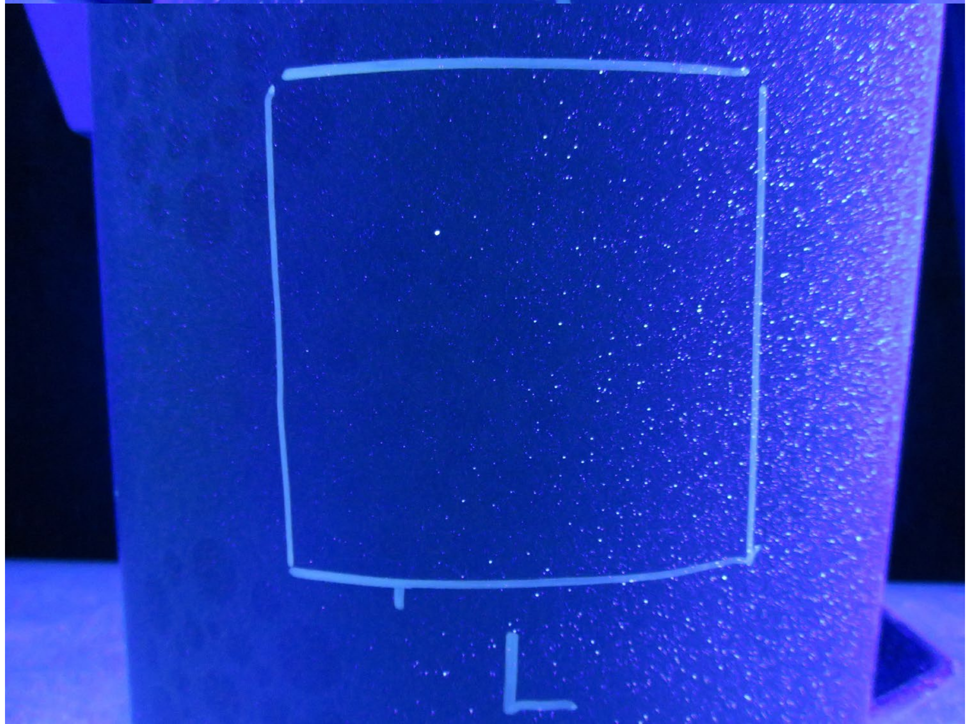

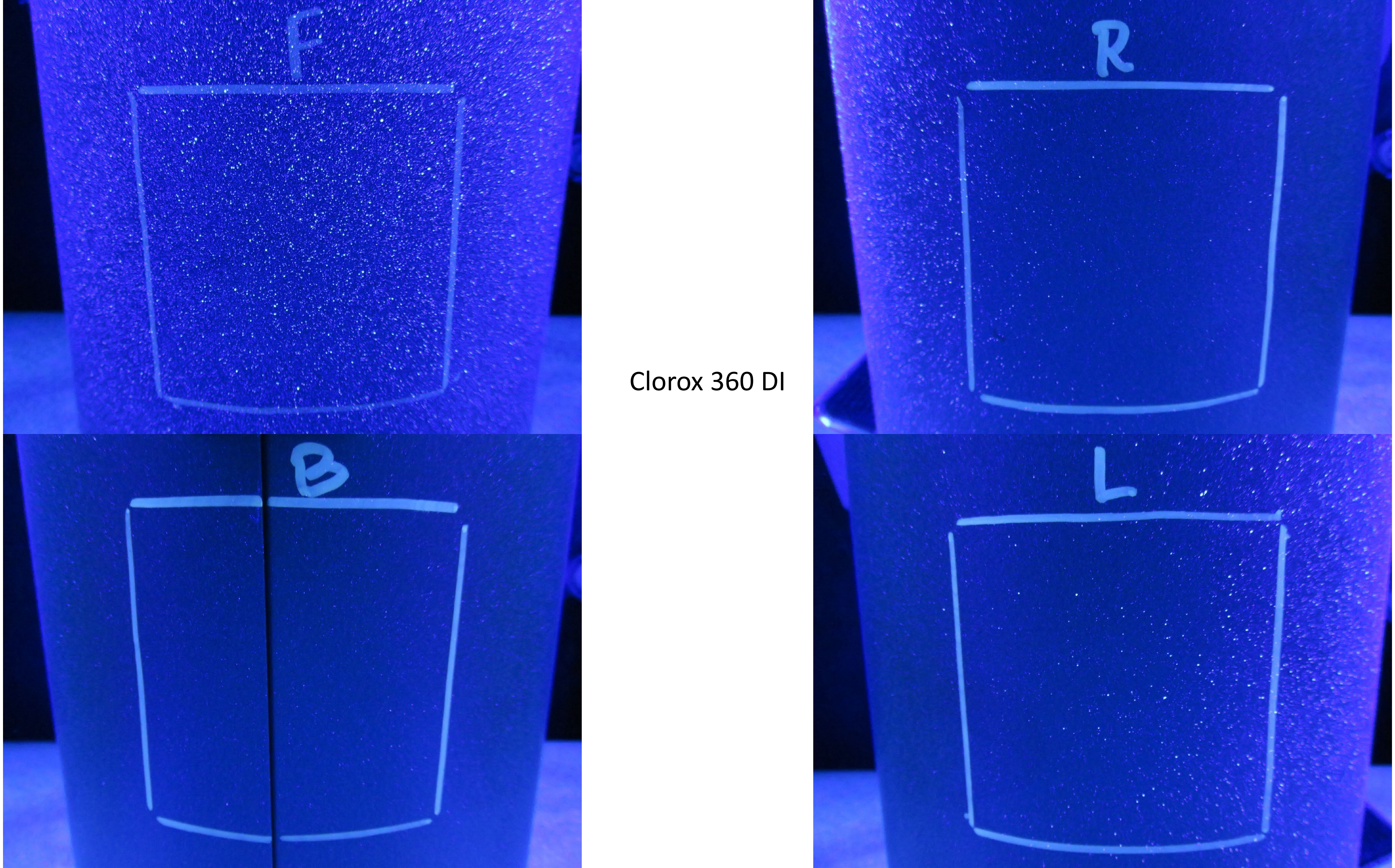


Clorox 360 DI

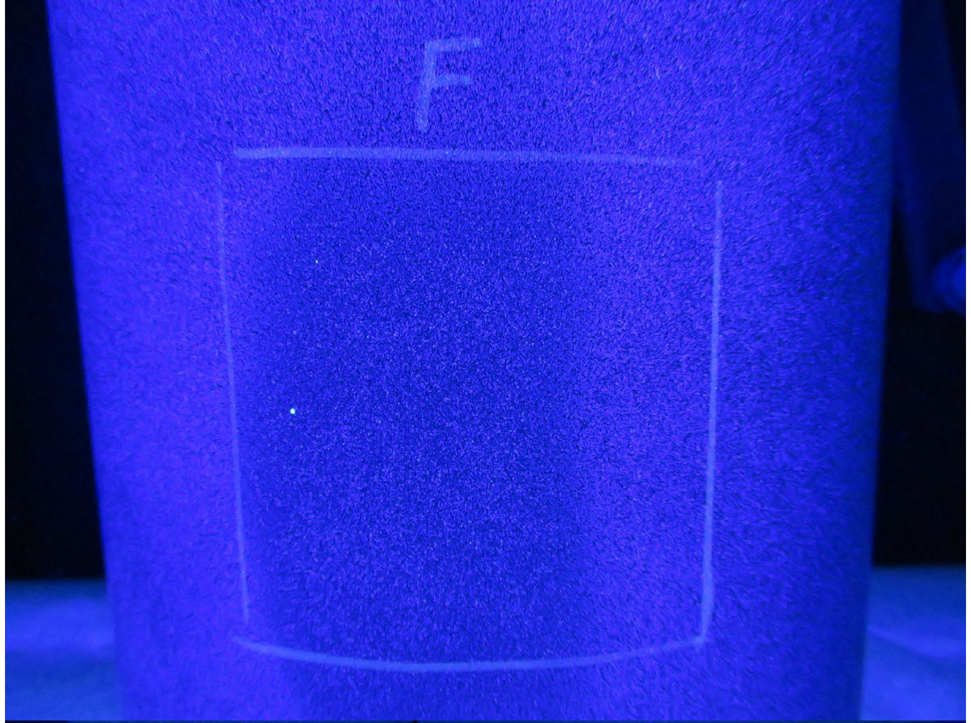


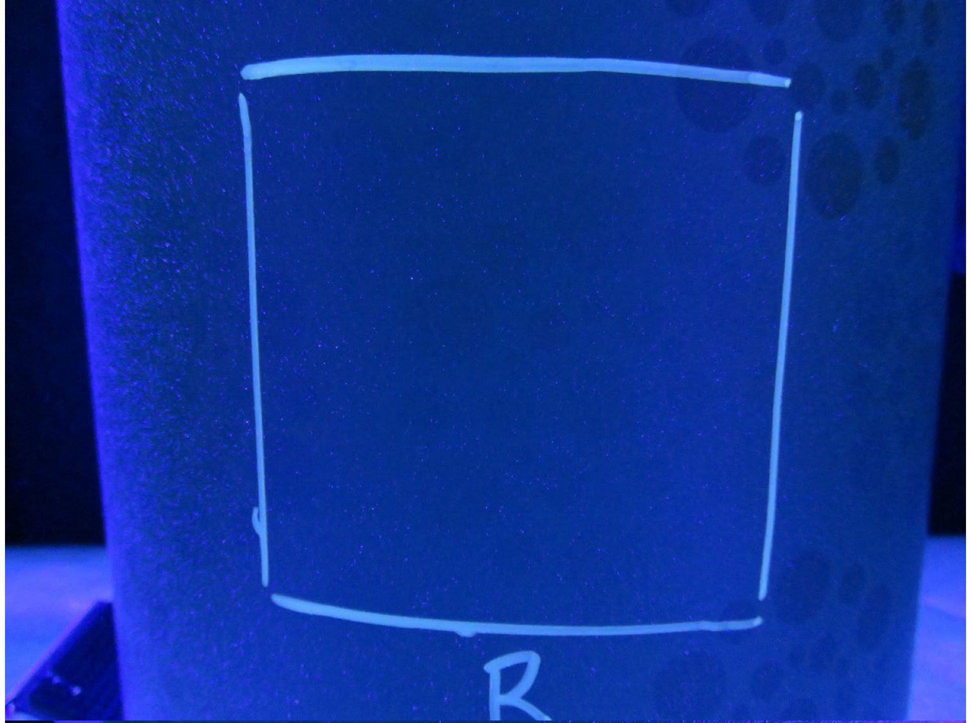


aerofog

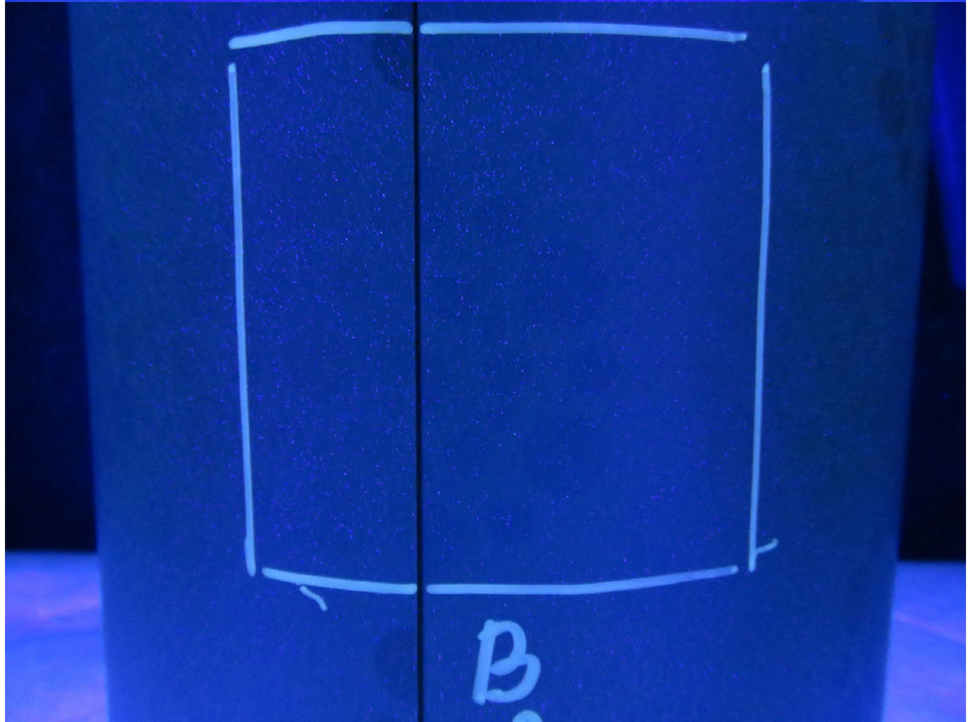


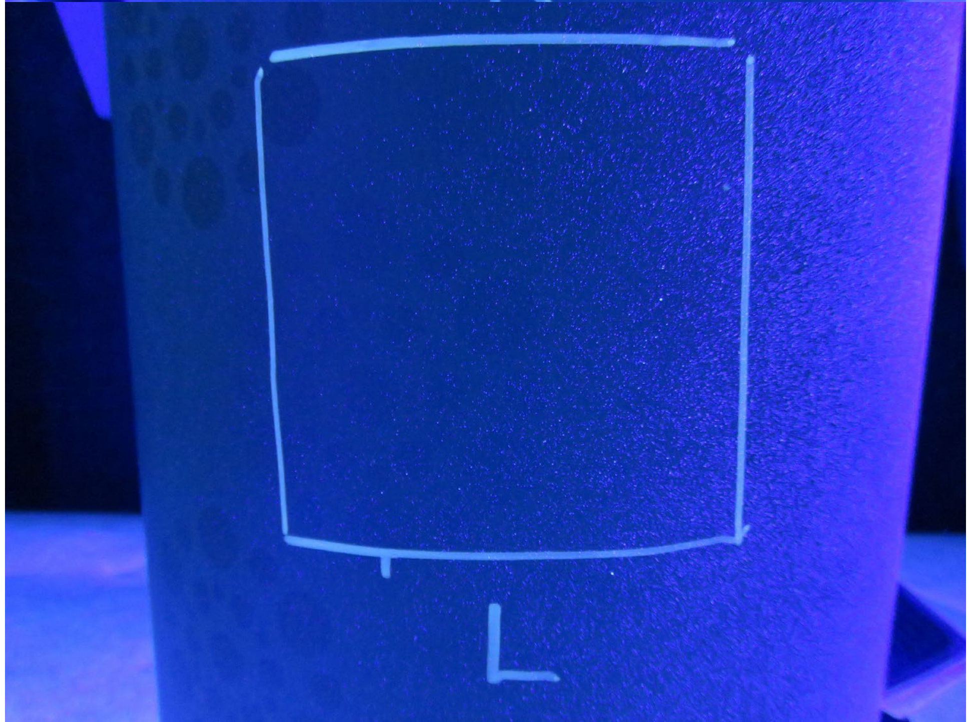

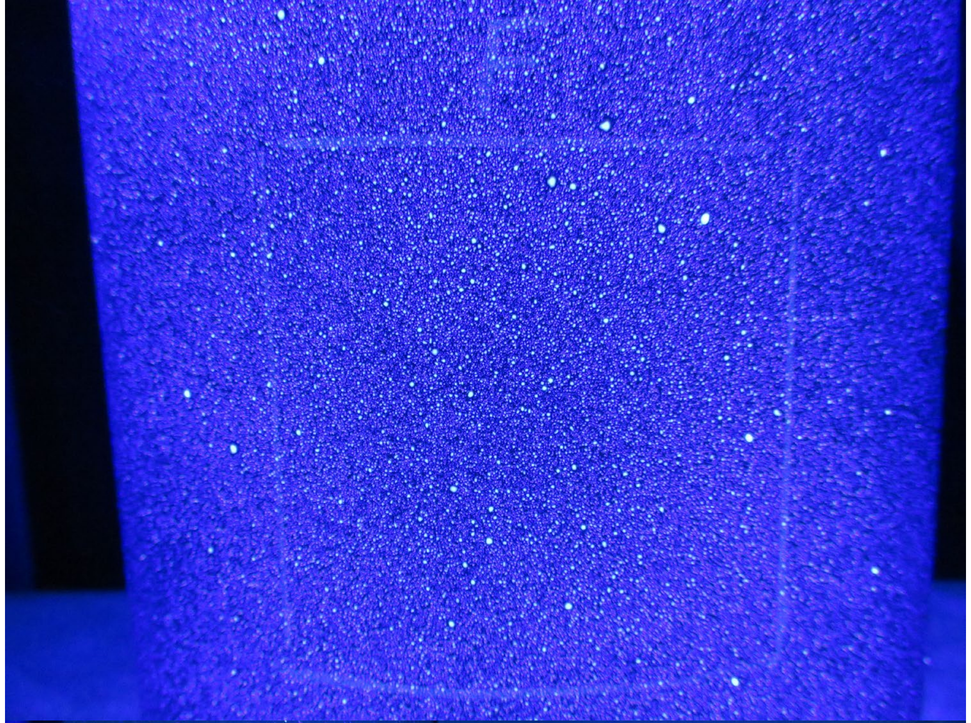

iphisius

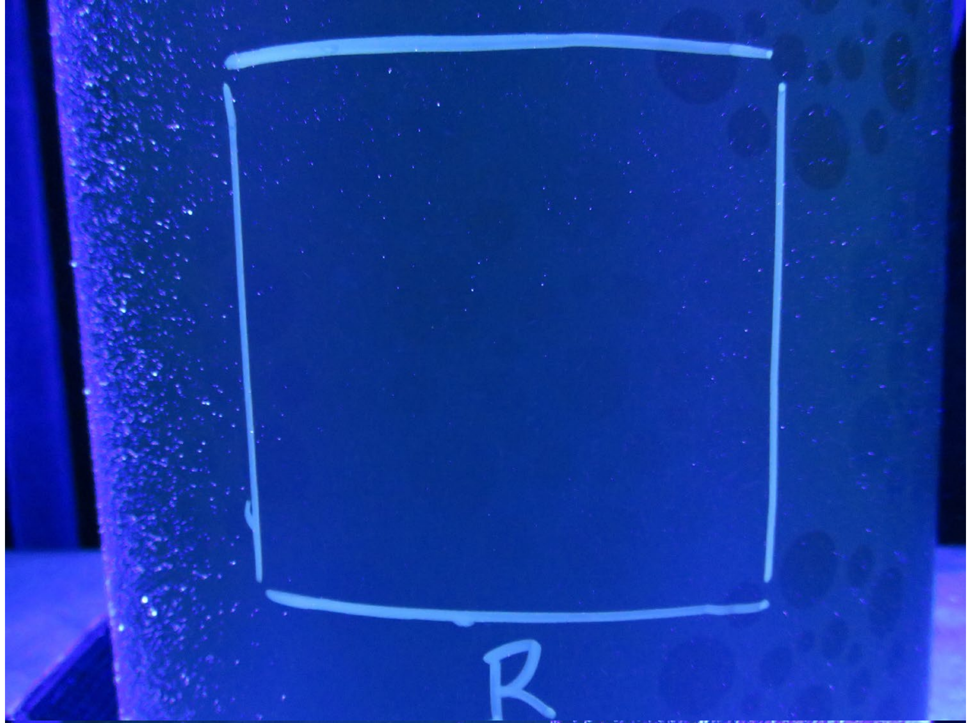


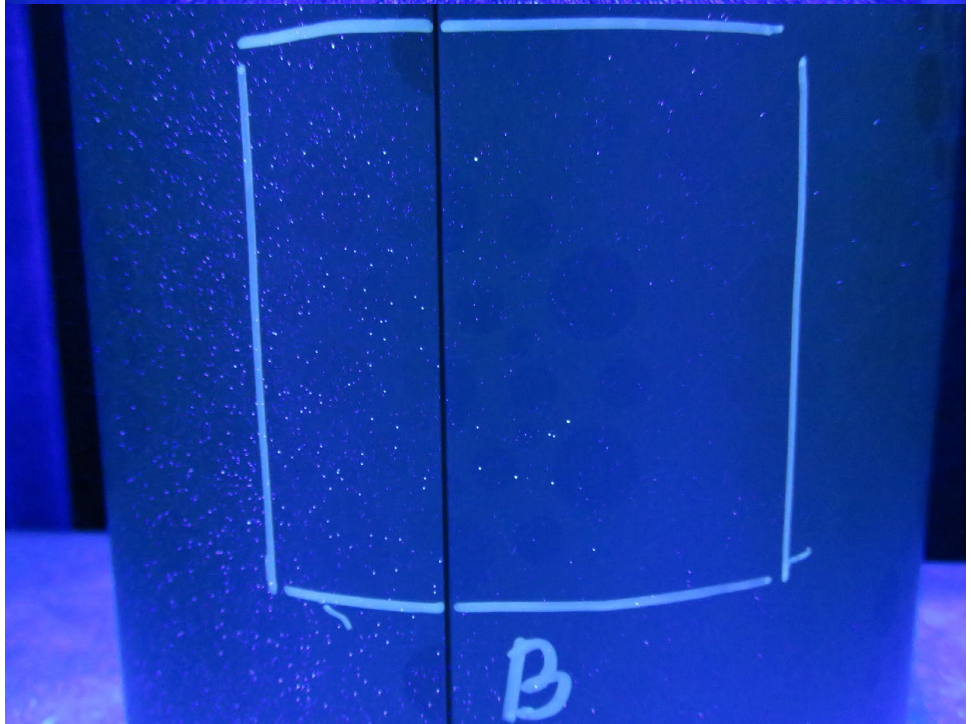


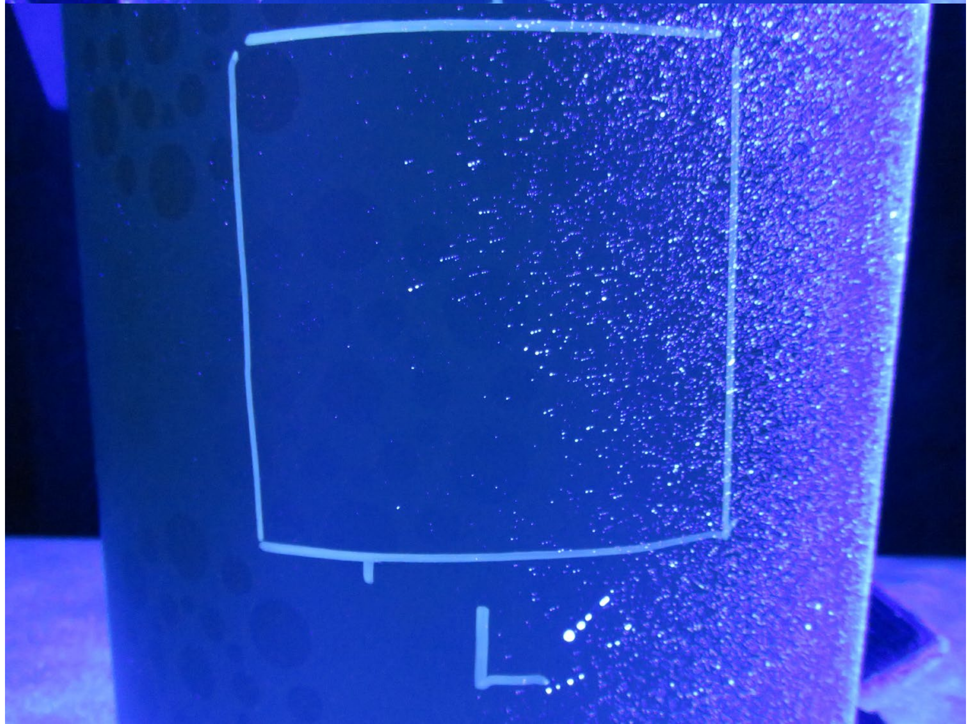

Clorox 360

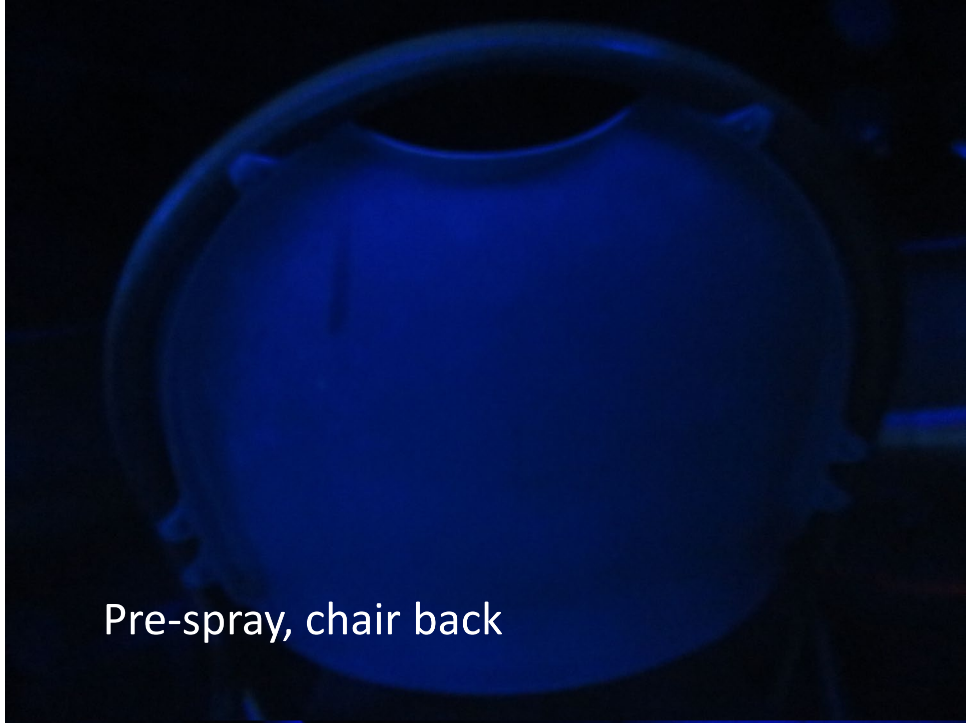
Pre-spray, chair back

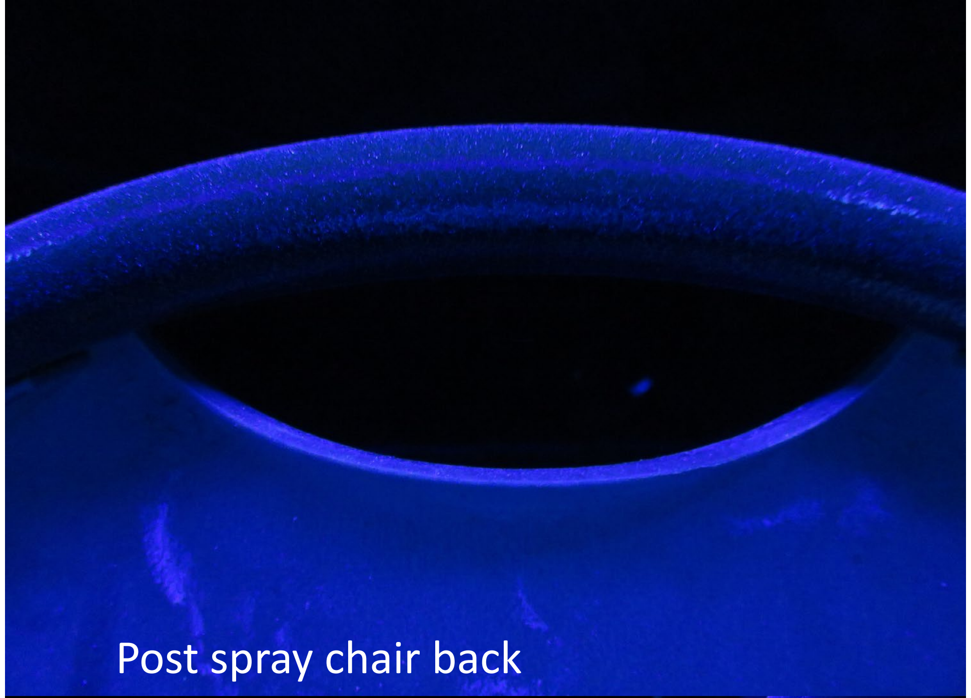
Post spray chair back

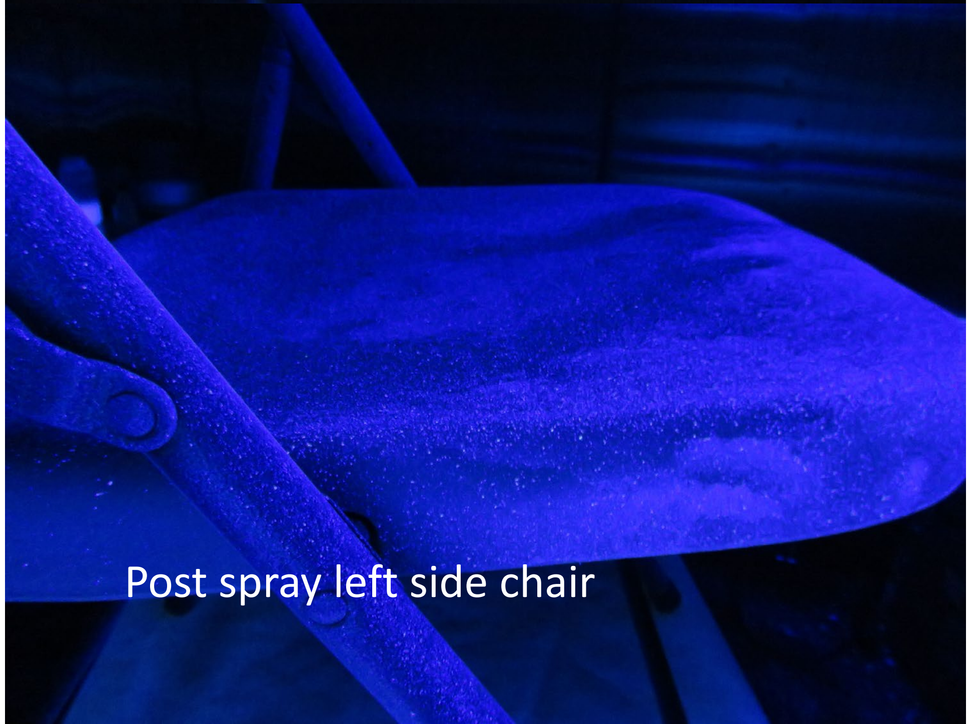
Post spray left side chair

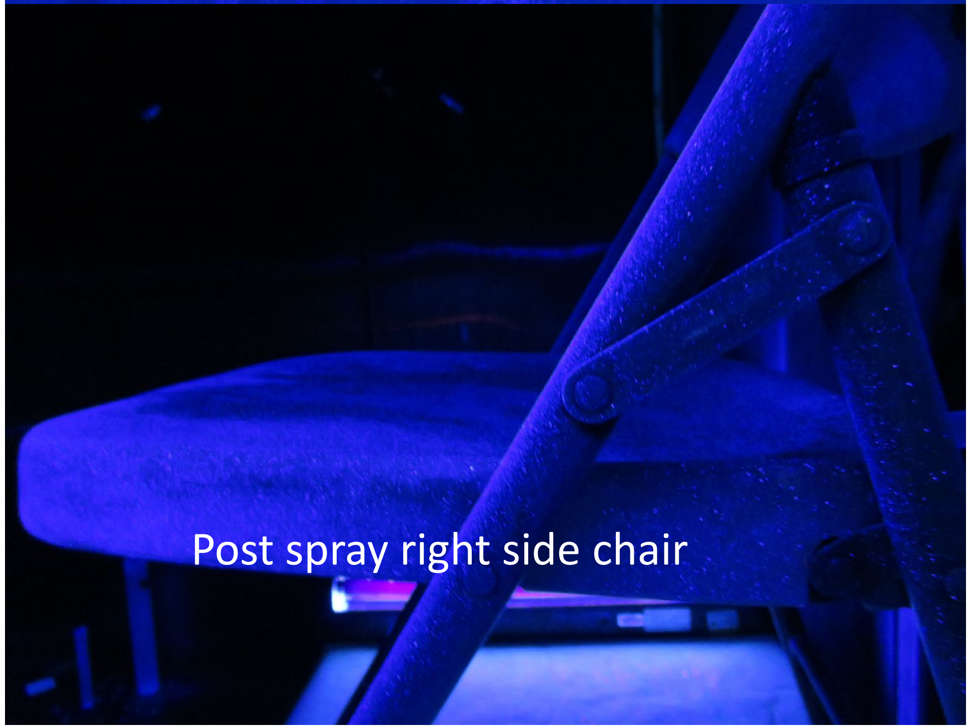
Post spray right side chair

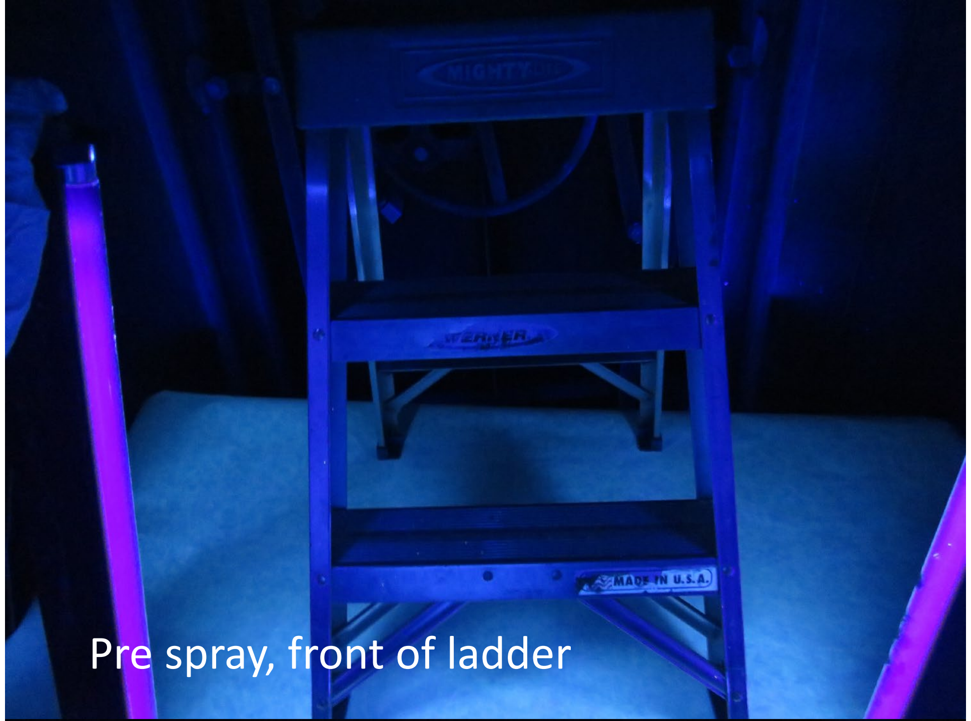

Pre spray, front of ladder

Clorox 360

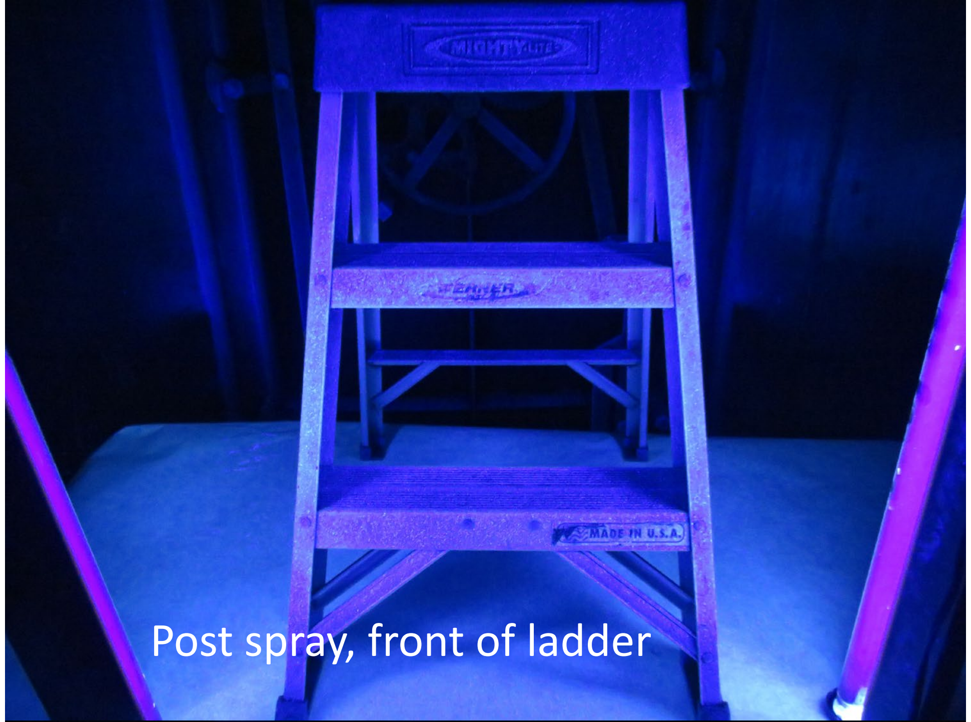

Post spray, front of ladder

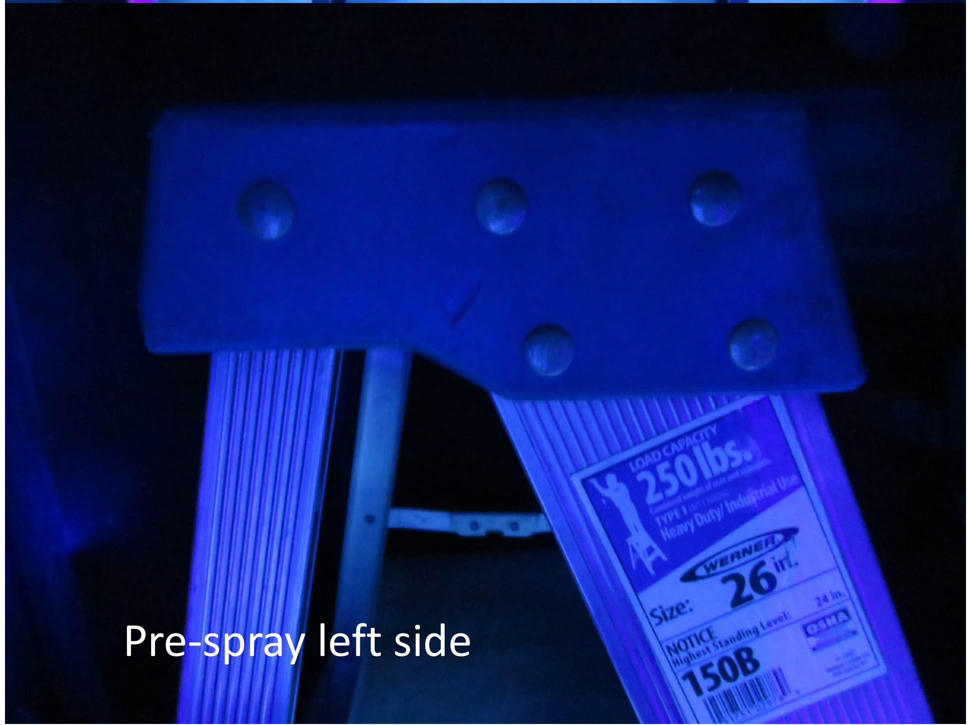

Pre-spray left side

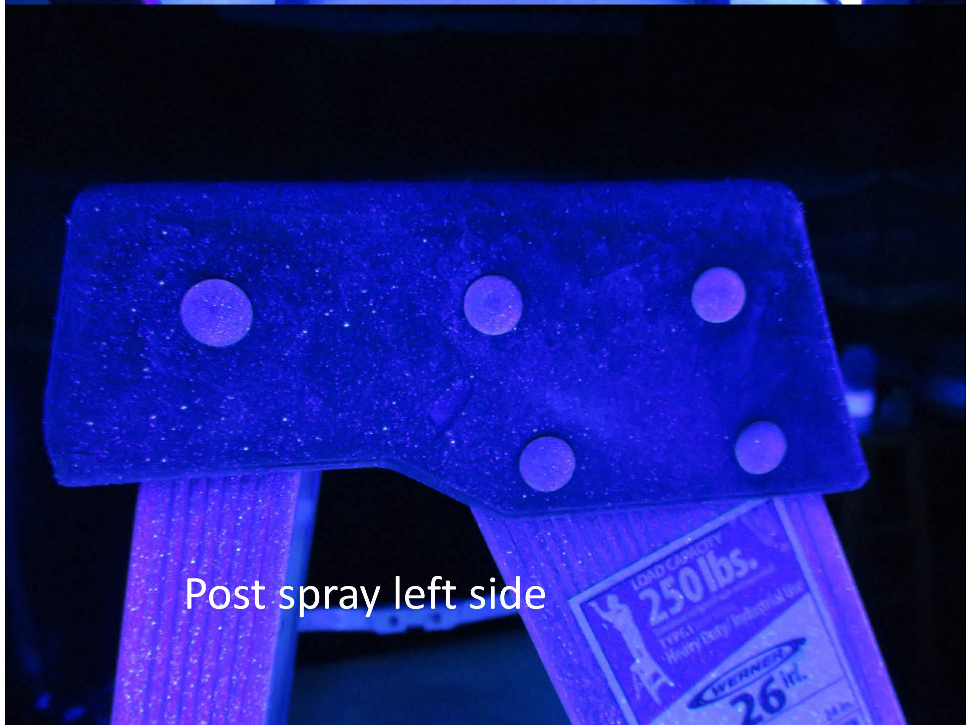

Post spray left side

Clorox 360

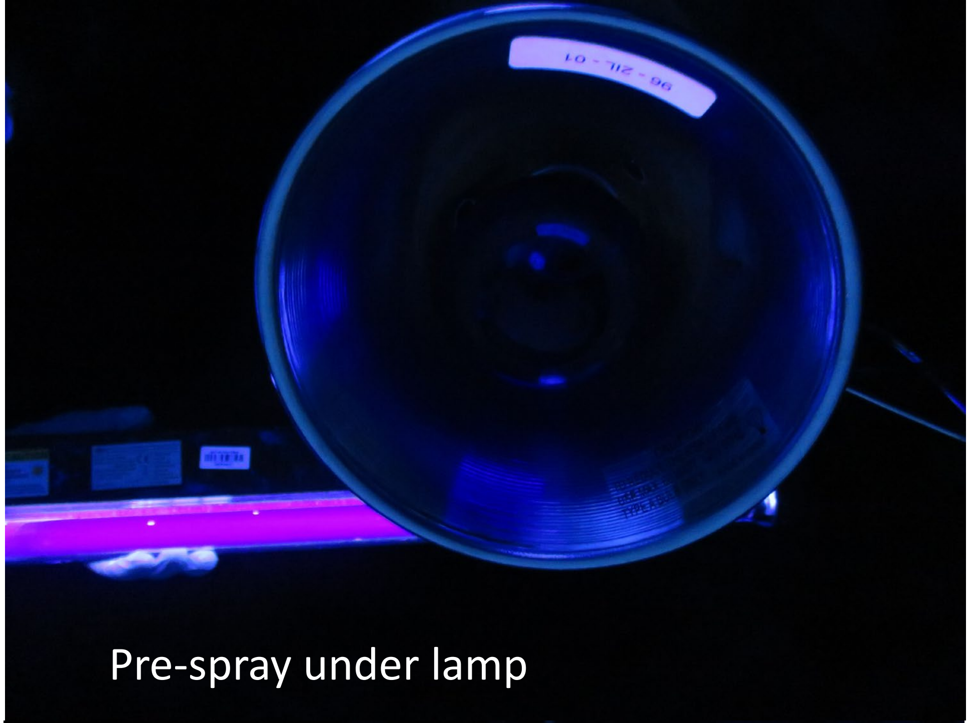

Pre-spray under lamp

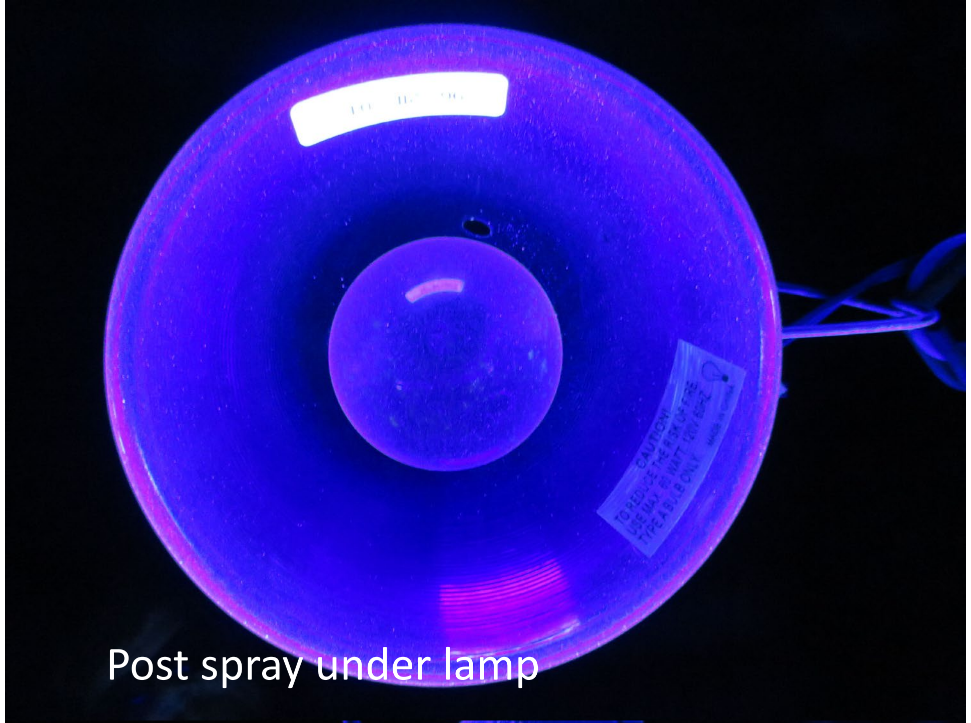

Post spray under lamp

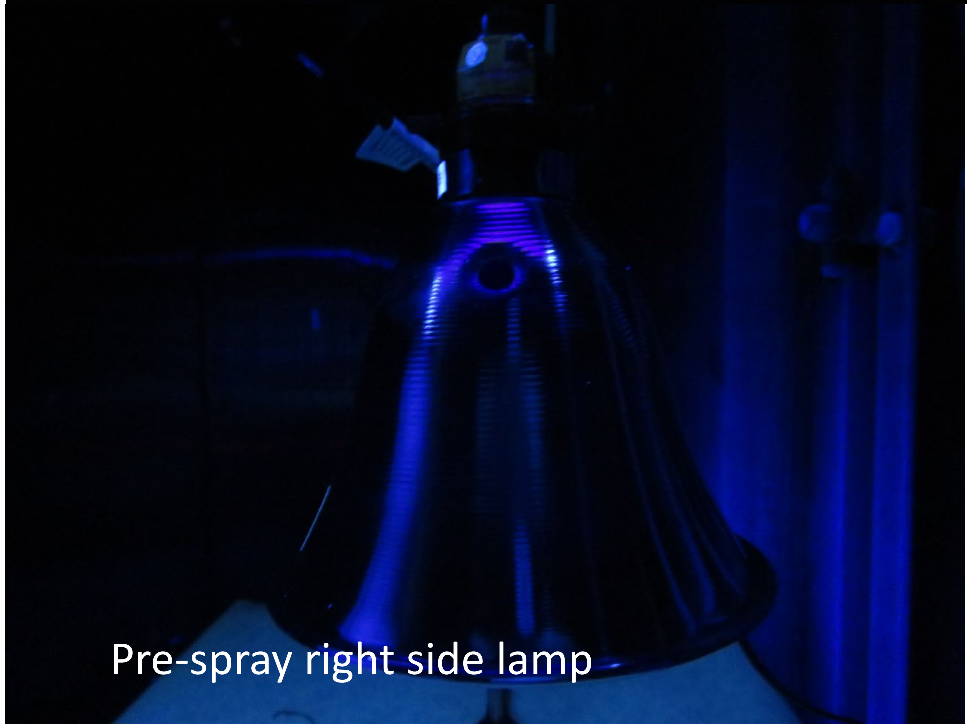

Pre-spray right side lamp

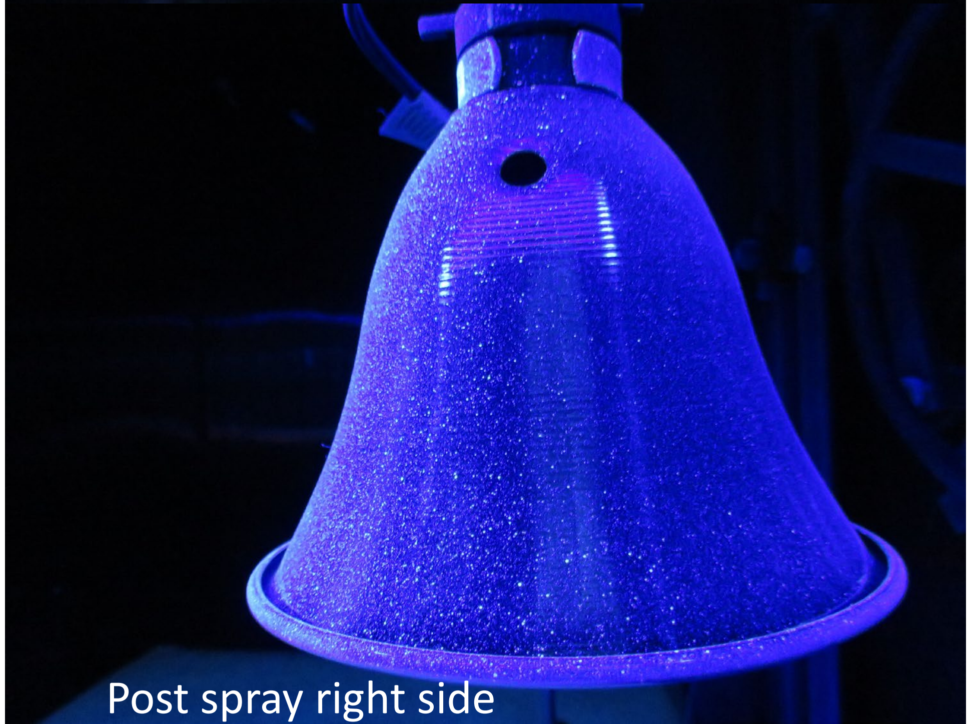

Post spray right side
