# Supplementary material for: Evaluation of electrostatic sprayers and foggers for the application of disinfectants in the era of SARS-CoV-2
Source: PLoS One. 2021 Sep 30;16(9):e0257434. doi: 10.1371/journal.pone.0257434 (PMC8483385; doi:10.1371/journal.pone.0257434)
Supplement: S3 File — (PDF) [file pone.0257434.s003.pdf]

Wetness tests

This test series was conducted to simply determine whether test materials remain wet for 10 minutes after spraying with water, how much water is initially deposited, and how much is lost to evaporation. (Ten minutes is a typical contact time for many disinfectants).

Test methods

Five ESS configurations were evaluated by spraying water onto 14-inch by 14-inch coupons of either plastic, stainless steel, or glass. The coupons were oriented in both a horizontal and vertical position. Spray distances varied according to the sprayer, based on manufacturer recommendations, and coupons were sprayed until droplets started to visually coalesce on the surface. Temperature in the test chamber was controlled to approximately 21 °C and relative humidity was controlled to approximately 35%; air flow in the test chamber was approximately 1 m/s.

For each material/sprayer/coupon orientation combination, one positive control coupon was used, and three test coupons were used. For the positive controls, the coupon was sprayed and then immediately wiped dry with a laboratory tissue. The wipe was weighed before and after collecting the water from the positive control, to determine the initial mass of water deposited onto the coupon. For the test coupons, a wipe was also used but 10 minutes after spraying. The mass of water collected from the positive controls was then compared to the mass of water remaining after 10 minutes, to determine how much water had evaporated.

Wetness test results

The results for the wetness tests are summarized in Figure 1 and show the amount of water recovered from the test coupons initially after spraying (the positive controls) and after 10 minutes of drying time. The results are averaged across the five sprayer configurations tested, for the coupon orientation (horizontal or vertical) and material.

Although test materials were sprayed until droplets began to coalesce (a somewhat subjective determination), materials in the horizontal orientation generally had higher amounts of water initially deposited than when coupons were oriented vertically (presumably due to runoff). After the initial deposition results are converted to more typically reported units, average results for the vertical coupons ranged from 23-33 ounces per 1000 ft^2, and average results for the horizontal coupons ranged from 38-65 ounces per 1000 ft^2. The deposition results are generally consistent with manufacturer-recommended spray deposition quantities (see Table 1).

The percent water loss after 10 minutes for coupons in the vertical orientation ranged from 78-95%, while the loss of water for coupons in the horizontal position ranged from 65-81%. In both coupon orientations, the plastic material had the least amount of water loss. Out of a total of 90 test coupons, 12 were completely dry after the 10-minute contact time, and the majority of these were in the vertical position. In a final note, visual inspection of the coupons after 10 minutes showed that drying on the surface was uneven, such that portions of the surface were visibly dry, in contrast to the method reporting remaining water mass.

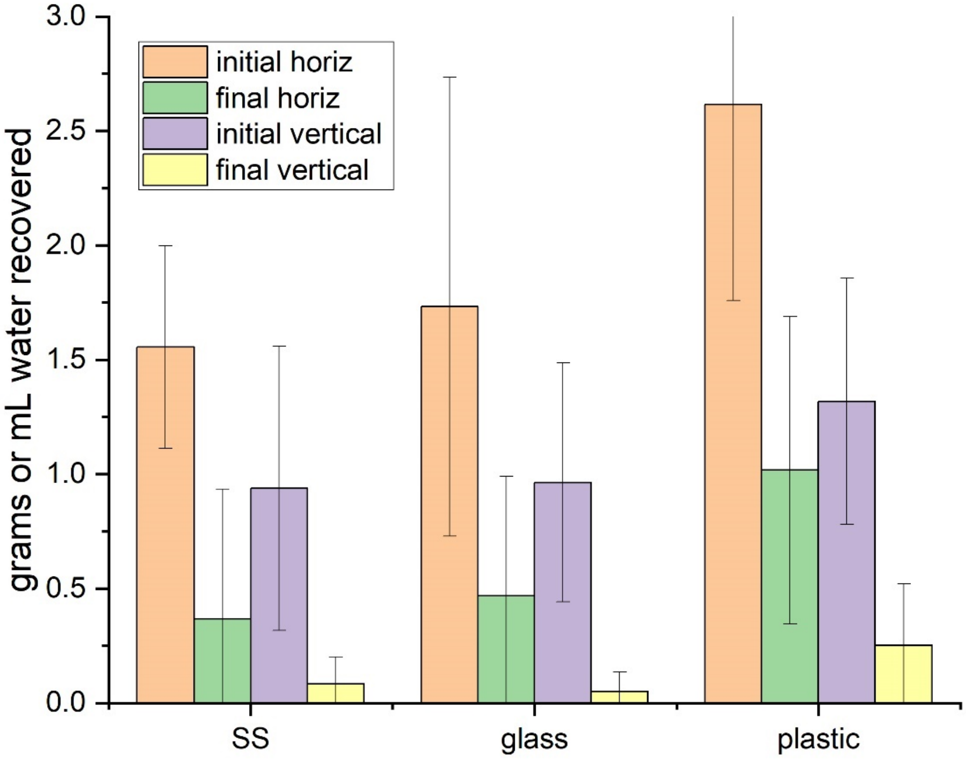

Figure 1. Average (± SD) amount of water (grams or milliliters) recovered from test coupons immediately after spraying (initial) or after 10 minutes (final), in either the horizontal or vertical position. SS= stainless steel.
